# Mycosporine-Like Amino Acids: Potential Health and Beauty Ingredients

**DOI:** 10.3390/md15100326

**Published:** 2017-10-21

**Authors:** Ewelina Chrapusta, Ariel Kaminski, Kornelia Duchnik, Beata Bober, Michal Adamski, Jan Bialczyk

**Affiliations:** 1 Department of Plant Physiology and Development, Faculty of Biochemistry, Biophysics and Biotechnology, Jagiellonian University, Gronostajowa 7, 30-387 Krakow, Poland; ariel.kaminski@uj.edu.pl (A.K.); kornelia.zabaglo@uj.edu.pl (K.D.); beata.bober@uj.edu.pl (B.B.); michal.adamski@doctoral.uj.edu.pl (M.A.); j.bialczyk@uj.edu.pl (J.B.); 2 Institute of Botany, Faculty of Biology and Earth Sciences, Jagiellonian University, Kopernika 27, 31-501 Krakow, Poland

**Keywords:** MAAs, ultraviolet radiation, UV-absorbing compound, sunscreen, erythema, melanoma, photoaging, skin renewal, biomaterial

## Abstract

Human skin is constantly exposed to damaging ultraviolet radiation (UVR), which induces a number of acute and chronic disorders. To reduce the risk of UV-induced skin injury, people apply an additional external protection in the form of cosmetic products containing sunscreens. Nowadays, because of the use of some chemical filters raises a lot of controversies, research focuses on exploring novel, fully safe and highly efficient natural UV-absorbing compounds that could be used as active ingredients in sun care products. A promising alternative is the application of multifunctional mycosporine-like amino acids (MAAs), which can effectively compete with commercially available filters. Here, we outline a complete characterization of these compounds and discuss their enormous biotechnological potential with special emphasis on their use as sunscreens, activators of cells proliferation, anti-cancer agents, anti-photoaging molecules, stimulators of skin renewal, and functional ingredients of UV-protective biomaterials.

## 1. Introduction

Ultraviolet radiation (UVR) is the part of the solar electromagnetic spectrum with a wavelength ranging from 200 to 400 nm. Based on its physical properties and biological activity, UVR is divided into three bands: UV-A (320–400 nm), UV-B (280–320 nm) and UV-C (200–280 nm). Nevertheless, UVR reaching the Earth’s surface represents only a small portion of the entire UVR emitted by the sun and is mainly composed of wavelengths above 290 nm (UV-A with a small UV-B component up to 10%). The remaining short-wavelength part of the UV-B spectrum (90%) and the entire UV-C spectrum usually do not penetrate the Earth’s stratosphere as they are absorbed by the ozone layer [1,2]. The incident UV rays are considered to be the main harmful environmental factor for living beings [3]. UVR is damaging to a wide variety of biological systems because short waves with high frequencies are extremely energetic. UV-A is ubiquitous and is present during the entire year, and its intensity is constant regardless of season and weather conditions. The acuteness of UV-B is the highest during the summer months in the 4-hour period around solar noon and under a clear sky [4,5,6]. Moreover, since the late 1970s, the progressive depletion of the ozone and changes in its permeability have been observed, and these changes have contributed to a marked increase of UV-B amount reaching the Earth’s surface [4,7]. These changes are primarily a consequence of rapid industrialization that increases the level of anthropogenic air pollution [7,8]. As a result, the intensity of UV-B can be up to 1.5 W·m^−2^ in the temperate zone or up to 2 W·m^−2^ in the equatorial zone, whereas the intensity of UV-A and visible light (400–700 nm) is approximately 50–60 W·m^−2^ and 500 W·m^−2^, respectively [8]. These outcomes are not satisfactory, especially now when the incidence rates of various human skin disorders are dramatically escalated each year [9,10,11]. Therefore, the issue of ensuring adequate fully safe protection for human beings to reduce the risk of sun exposure is crucial in the coming years. Currently, a lot of research focusing on exploring the novel, highly efficient natural UV-absorbing compounds that could be used as active ingredients in sun care products to protect human skin is inspired by the resourceful defense strategies developed by various terrestrial and marine organisms to minimize UV-induced damage [12,13,14].

In this review, we cover the group of relevant UV-protective mycosporine-like amino acids (MAAs) and summarize (1) their detailed structure and physicochemical properties; (2) their biosynthetic pathways and regulation; (3) the current state of knowledge of their ecological functions with special emphasis on the photoprotective role; (4) their occurrence, distribution, and natural sources; (5) their application potential as bioactive substances. The primary aim of this article is to update and complete the information described in previous reviews on MAAs with particular emphasis to their biotechnological and industrial potential [3,12,15,16,17,18,19,20].

## 2. Mycosporine-Like Amino Acids

Recently, mycosporine-like amino acids (MAAs) have attracted increasing research interest. They belong to a family of secondary metabolites produced by a wide range of different organisms, especially those inhabiting ecosystems with a high concentration of sunlight, such as marine and freshwater environments, to protect against solar radiation [12,16,21,22,23,24]. The history of research on MAAs dates back to the late 60s of the last century [25]. Since the discovery of MAAs, knowledge including information on their structure, properties, functions, and distribution is constantly developing.

### 2.1. Structure and Physicochemical Properties

**Figure 1:**
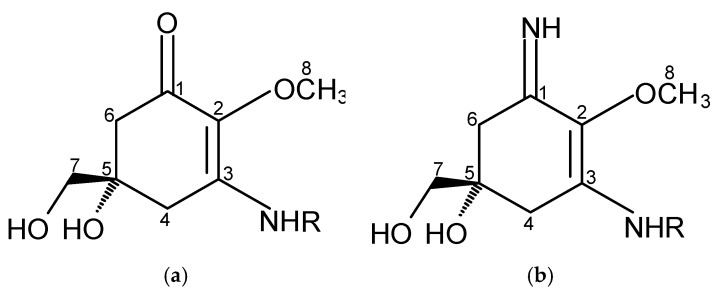
(**a**) Aminocyclohexenone and (**b**) aminocyclohexeniminone rings.

MAAs are of low molecular weight (generally < 400 Da), colourless, uncharged, water-soluble ampholytes, and they share the same chemical structure but differ in the substituents and/or presence of amino acids. They are composed of a cyclohexenone or cyclohexenimine chromophore with the nitrogen substituent (Figure 1) [26,27,28].

**Figure 2:**
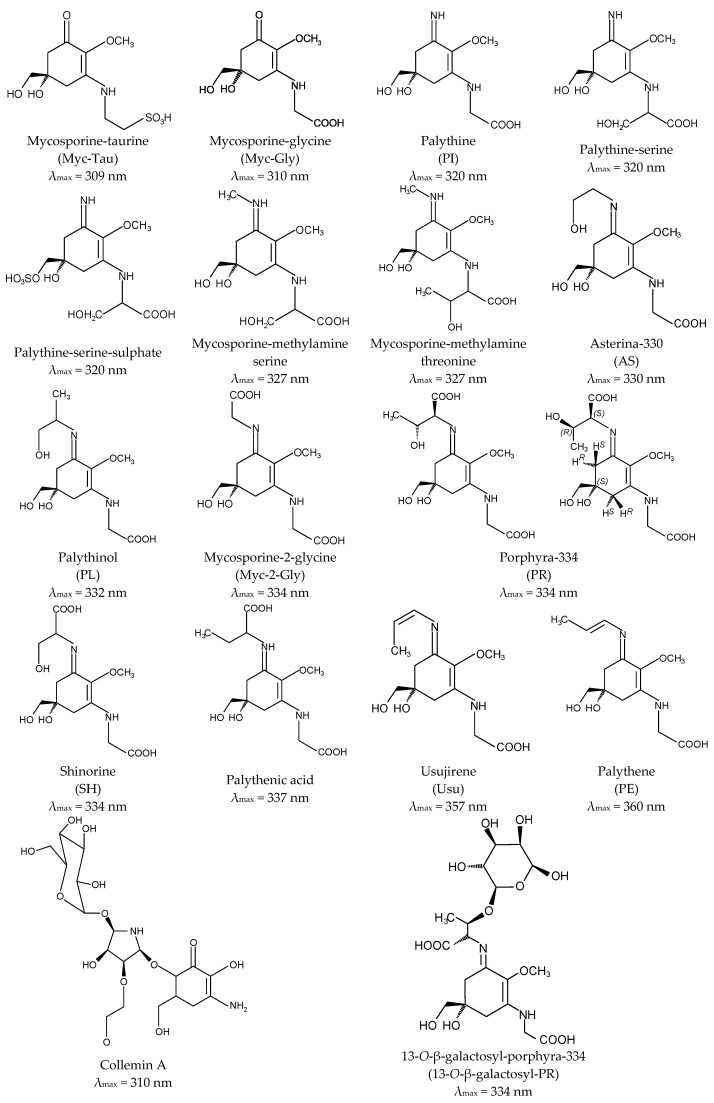
Chemical structure of selected MAAs with their absorption maxima (λ_max_).

Aminocyclohexenone derivatives possess a cyclohexenone conjugated with an amino acid. This group includes e.g., mycosporine-glycine (Myc-Gly), mycosporine-taurine (Myc-Tau), mycosporine-alanine, mycosporine-serine, mycosporine-serinol, mycosporine-glutamicol, mycosporine hydroxylglutamicol, mycosporine-glutamine, mycosporine-glutaminol and collemin A (Figure 2) [15,29]. Aminocyclohexenimines are represented by e.g., shinorine (SH), porphyra-334 (PR), usujirene (Usu), asterina-330 (AS), palythine (PI), palythinol (PL), palythene (PE), mycosporine-2-glycine (Myc-2-Gly), mycosporine-glutamic acid-glycine, mycosporine-glycine-valine, catenelline, euhalothece-263, aplysiapalythine A, B, C and 13-*O*-β-galactosyl-porphyra-334 (13-*O*-β-galactosyl-PR) (Figure 2) [30,31,32]. Typically, each cyclohexenimine ring backbone contains the glycine attached to the 3rd carbon atom and an additional amino acid or amino alcohol or enaminone chromophore to the 1st carbon atom. Also, the glycine may be substituted by methylamine [33], and within the imine group may occur glycosidic bonds or sulphate esters [34,35].

Differences in the structure of these compounds, namely in the type of ring and substituents, determine their specific absorption spectra. The outstanding characteristic of all MAAs is their ability to absorb UV radiation in the harmful range from 309 to 362 nm and a high molar absorptivity (*ξ*) from 2.81 × 10^4^ to 5.00 × 10^4^ M^−1^·cm^−1^ [1,16,22,24,36,37,38,39,40,41,42]. The oxo-mycosporines exhibit absorption maxima in the UV-B region, and imino-mycosporines in the UV-A region [43]. Myc-Gly is the primary MAA and is converted to the imino-mycosporines via chemical or biochemical modifications [44,45,46,47]. Increased level of oxygen stimulates these transformations because the absorption of UV-A effectively prevents the UV-A-induced formation of oxygen free radicals. The ketone group in the Myc-Gly molecule is replaced by a nitrogen atom, which leads to a shift in the absorption spectrum to the UV-A fraction [45,48,49]. MAAs are widely regarded as the most effective UV-A-absorbing compounds in nature [50]. A full understanding of their stability under the influence of different physicochemical stressors is still far from being complete and the literature data clearly indicate that there is no common pattern for all MAAs. For example, Myc-Gly is highly resistant to various pH conditions up to 24 h [51], whereas exposed to 80 °C converts to β-diketone (6-deoxygadusol) and glycine within 3 h [52]. In turn, PR in solutions of pH from 1 to 11 remains stable up to 24 h and rapidly degrades at extremely high pH 12 and 13 [53]. The process of its degradation is correlated with an increase in temperature up to 60 °C and 100 °C, regardless of the pH [53,54], and at 120 °C dehydrates to a derivative with a high antioxidant potential [55].

### 2.2. Biosynthetic Pathways and Their Regulation

**Figure 3:**
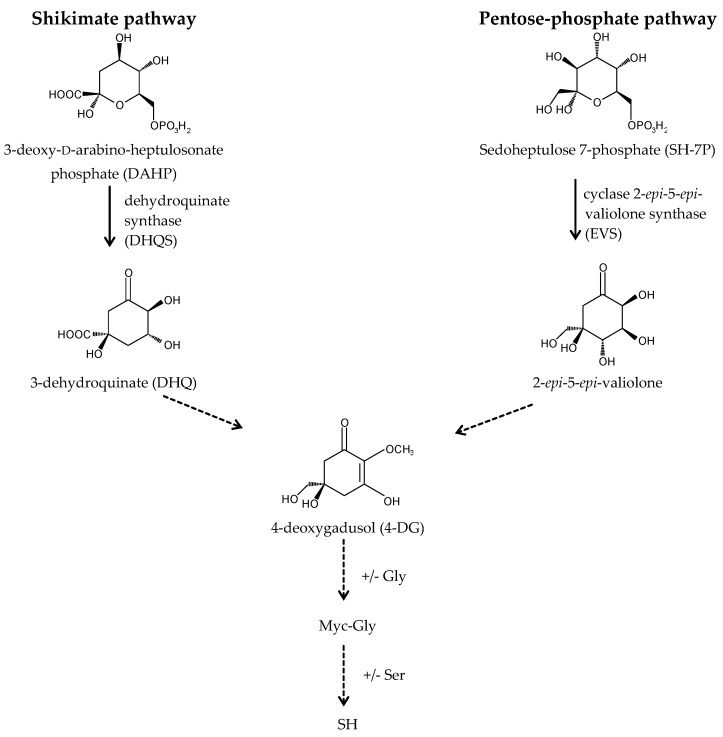
Proposed MAAs biosynthetic pathways.

A long-standing assumption suggests that MAAs biosynthesis takes place through the first branch of the shikimate pathway (Figure 3) [22,39,56]. The core of MAAs, derived from 3-dehydroquinate (3-DHQ), is converted to the immediate precursor of MAAs, 4-deoxygadusol (4-DG), and then to cyclohexenones (gadusols) [16,22,27,56,57,58]. This assumption is supported by an experiment showing that the production of MAAs in the coral *Stylophora pistillata* is blocked after the application of glyphosate, a specific shikimate route inhibitor [56]. Singh et al. (2010b) proposed that two genes, YP_324357 (Ava_3857) and YP_324358 (Ava_3858), encoding the enzymes of this route, the O-methyltransferase (O-MT) and the dehydroquinate synthase (DHQS), respectively, are involved in the process of MAAs synthesis in the cyanobacterium *Anabaena variabilis* PCC 7937 [59]. Recently, the assumption that MAAs are synthesized by the shikimate route has been challenged. Research conducted by Balskus and Walsh (2010) on *A. variabilis* PCC 7937 shed new light on this process [60]. Their findings suggest that MAAs biosynthesis occurs via the pentose-phosphate pathway (Figure 3). MAAs originate from the intermediate, sedoheptulose-7-phosphate (SH-7-P), produced by the pentose phosphate route and an adenosine triphosphate (ATP)-dependent enzymatic imine formation through a four-enzyme pathway. Their study confirmed that the primary MAA, SH, can be synthesized by a four-step pathway encoded by a gene cluster containing DHQS, O-MT, ATP-grasp and the nonribosomal peptide synthetase (NRPS) homolog. DHQS YP_324358 and O-MT YP_324357 convert SH-7-P into 4-DG, then ATP-grasp homologue YP_324356 (Ava_3856) converts 4-DG and Gly into Myc-Gly, and finally NRPS-like enzyme YP_324355 (Ava_3855) attaches Ser to Myc-Gly to create SH. The cluster of genes corresponding to the SH biosynthetic route has been confirmed in cyanobacteria and other organisms, such as fungi, sea anemones, dinoflagellates (in chloroplasts) and corals (in sperm) [60,61]. Generally, MAAs biosynthetic pathways are not specific for each species and depend on the several abiotic conditions, which stimulate their course [62,63,64]. MAAs biosynthesis is regulated in particular by the spectral distribution and intensity of solar radiation [39]. Blue light in the photosynthetically active radiation (PAR) spectrum and UV-A boost the production of MAAs in free-living dinoflagellates [36,65,66,67,68] and Antarctic diatoms [69,70,71]. UV-A is the most important wavelength range to induce the synthesis of SH and PI in the red macroalga *Chondrus crispus* [72,73], whereas UV-B has the most pronounced effect to enhance the accumulation of MAAs in cyanobacteria [74,75]. In turn, MAAs synthesis in corals requires a combination of three wavelength ranges, UV-B, UV-A and PAR [56,76]. The conversion of Myc-Gly into the secondary MAAs, PR and SH, in *A. doliolum* occurs under combined UV and PAR irradiation [77]. Furthermore, MAAs concentration and composition are correlated with the intensity of UVR [78]. Therefore, MAAs accumulation decreases with the increasing depth of reservoir. In addition, the highly UV-B-stressed cells of the dinoflagellate *Alexandrium tamarense* synthesize more secondary MAAs, while less-stressed cells contain more primary MAAs [67]. The regulation of MAAs production involves also other abiotic factors, such as desiccation, temperature, salinity, and nutrients availability, which can affect alone or in combination with UVR [22,74,79,80]. MAA levels in the soft corals *Sinularia flexibilis* and *Lobophytum compactum* have been shown to be up-regulated under the thermal stress as well as during the simultaneous exposure to UVR [81]. In contrast, the cyanobacterium *Chlorogloeopsis* PCC 6912 exposed to the increased temperature or cold shock does not demonstrate the formation of MAA; only UVR and salt stress induce its biosynthesis [82]. Research confirms that nitrogen (N) is an essential element for MAAs synthesis. High concentrations of ammonium significantly promote their accumulation in the red alga *Porphyra columbina* [83] and *A. variabilis* PCC 7937 either with or without UV irradiation [74]. An opposite effect was observed in two dinoflagellates *Gymnodinium* cf. *instriatum* and *Akashiwo sanguinea* under nitrate limitations [84]. Moreover, nitrogen availability can influence the composition of MAAs. The N-starved cells of *A. sanguinea* have a much higher concentration of the primary MAA, Myc-Gly, containing only one atom of nitrogen [84]. In turn, phosphate depletion in the dinoflagellate *Glenodinium foliaceum* cultures can stimulate the synthesis of secondary MAAs, SH, PI and AS [85], and sulphur deficiency can regulate the production and conversion of Myc-Gly into the secondary MAAs in the *A. variabilis* PCC 7937 [86]. However, the conclusions regarding the biosynthetic pathway of MAAs remain unclear, and its detailed identification will help provide a better understanding of the function of MAAs in response to the environmental stress factors.

### 2.3. Occurrence and Distribution in the Environment

To survive, nearly all organisms have developed a variety of natural defense strategies to counteract the adverse effects of UVR exposure, including biosynthesis of UV-screening compounds such as MAAs [16,37,44,45,56,87,88,89,90,91]. MAAs are widely distributed in the environment [92]. For the first time, their presence has been detected in cyanobacterium and several species of corals from the Great Barrier Reef [25]. Currently, MAAs family consists of more than 33 compounds [30], but obviously, further comprehensive studies using more sensitive techniques, such mass spectrometry, can reveal the existence of other MAA forms [33,93]. Reports have indicated that many taxonomically various groups of marine and terrestrial organisms from tropical latitudes to polar regions have evolved the ability to synthesize, accumulate and metabolize MAAs [1,22,23,37,39,44,89,94,95]. MAAs are present particularly in organisms living in a high UVR environment. The de novo synthesis ability of MAAs was demonstrated in marine heterotrophic bacteria, cyanobacteria, micro- and macroalgae, fungi, and lichens [22,23,95]. Furthermore, MAAs have been identified in bodies of some marine multicellular organisms (e.g., sponges, corals, sea stars, sea urchins, and fishes) despite the absence of the shikimate pathway in their cells [22,23,24,40,95,96,97,98,99,100,101]. It is assumed that animals can absorb MAAs from their algal food by diet transfer or that they can acquire them through symbiosis with algae and cyanobacteria or as a result of bacterial associations [22,23,24,96,97], and then subsequently accumulate them or intraconvert to other MAAs forms to function as photoprotective molecules [81,102]. Nevertheless, in a recent paper Osborn et al. (2015) reported that fishes are able to synthesize de novo gadusol and similar pathways occur in amphibians, reptiles, and birds [103]. Moreover, even genetically modified yeast with the fish genes can produce and secrete gaduzol [103,104]. MAAs might be located in the ocular tissue, skin, especially on the dorsal surface, external mucus, eggs, reproductive tissues (ovaries) and digestive track of the tropical shallow water reef fish [22,24,98,103,105,106,107,108,109,110]. MAAs concentration and composition depend on several factors, including diet. Carnivores accumulate much lower MAAs levels than herbivores [107]. Generally, their concentration in the cells is not more than 1% of dry weight. MAA composition varies depending on the taxonomic group. SH, PI and AS are most typical in algae, invertebrates, and chordates, respectively [107]. Additionally, the simultaneous coexistence of several MAAs with different absorbance maxima in the UV-A and UV-B ranges has been identified. This phenomenon undoubtedly allows for more effective protective filter than the presence of just one of these compounds [1]. Thus far, the richest composition of MAAs was observed in the *S. pistillata* [44].

### 2.4. Functions

The accurate role of MAAs in living organisms is still under discussion. They are characterized by a high biological activity, which has important therapeutic and ecological significance. The majority of studies on MAAs are focused on their photoprotective capability. Nevertheless, they are multifunctional secondary metabolites that have many cellular functions, such as antioxidant activity, osmotic regulation, reproduction control, nitrogen reservoirs [22].

#### 2.4.1. Photoprotection

The most important function of MAAs is photoprotection [3,17,22,39], and they are commonly described as “microbial sunscreens”. These compounds protect the cell due to their ability to disperse the harmful UVR into heat energy that dissipates into the surroundings without forming reactive photoproducts [42,80,111]. Several physicochemical characteristics of MAAs including a strong absorption in UV-A and UV-B regions of solar radiation spectrum, high molar extinction coefficients and resistance to several abiotic stressors give a strong evidence in favour of MAAs as efficient photoprotective compounds [51,112,113,114]. MAAs also effectively block the UVR-induced creation of thymine dimers in vitro [115]. The photoprotective efficiency of MAAs depends on their position in the cell. These compounds have been found mainly in the cytoplasm of several cyanobacterial species which prevent three out of every ten photons from reaching sensitive cellular targets [116,117,118]. However, in the cyanobacterium *Nostoc commune*, MAAs are actively excreted and extracellularly accumulated, thus resulting in more effective protection against UVR [80,118,119]. The presence of MAAs in marine animals confirms their photoprotection function not only to producers but also even to herbivores and carnivores [120]. Detection of MAAs in the fossils also supports their protection function against the harmful effects of UVR in the early geological eras [121].

#### 2.4.2. Biological Antioxidant Molecules

Although the synthesis of MAAs occurs mainly in response to UVR, MAAs can perform other functions in addition to UV-protection. It is suggested that certain MAAs, namely Myc-Gly and Myc-Tau, exhibit a strong antioxidant activity by quenching the reactive oxygen species (ROS) [30,80,119,120,122,123,124,125]. Myc-Gly effectively reduces the amount of singlet oxygen formed under illumination by the endogenous photosensitizers, methylene blue and eosin Y, and suppresses the adverse effects of photosensitization (type II reactions) in biological systems, such as lipid peroxidation, inactivation of mitochondrial electron transport, and hemolysis of erythrocytes [122,123]. Its high antioxidant efficacy is likely related to the lower reduction potential and greater capability to donate electrons to stabilize and inactivate the free radicals [123]. The results of in vitro experiments have shown, however, that Myc-Gly and Myc-Tau have moderate antioxidant activity compared to 4-DG, the MAA precursor, which has a strong antioxidant property [126]. AS, in combination with PI, is also characterized by a high antioxidant activity, although significantly lower than Myc-Gly and ascorbic acid. Moreover, it effectively inhibits the oxidation of β-carotene, an indicator of protection of lipid peroxidation. Other imino-mycosporines such as SH and PR show less activity or its absence [127].

#### 2.4.3. Protection against Abiotic Stress Factors

It was observed that MAAs can enhance cellular tolerance to abiotic stress factors such as salinity, desiccation, and temperature [80,128,129]. A hypersaline environment can lead to dehydration of cells and accumulation of ROS, which results in the generation of oxidative stress. Thus, MAAs provide osmotic balance to the cells [80]. In this regard, MAAs are abundant in high-salt ecosystems and often called “osmotic solutes” or “compatible solutes”. Many microorganisms store MAAs in the intracellular space, contributing to the formation of an osmotic pressure inside the cell, thus relieving pressure from salt stress in a hypertonic environment. Freshwater organisms also tend to accumulate these substances but in much smaller concentrations. Additionally, it is believed that MAAs, which have been demonstrated to exist in cold aquatic ecosystems, can also act as osmoprotectants under freezing conditions [68,80]. MAAs also contribute to the increase of the resistance of organisms to conditions where water becomes a limiting factor. The high concentrations of glycosylated MAAs have been reported in the extracellular matrix or sheath around microorganisms. However, the presence of only those substances does not provide sufficient protection against drought stress [130]. Moreover, MAAs can play a significant role in counteracting the effects caused by high temperatures.

#### 2.4.4. Nitrogen Storage

MAAs are considered to be an intracellular nitrogen reservoir. MAA molecules contain one or two nitrogen atoms, which can be released by suitable mechanisms when necessary [80,83].

#### 2.4.5. Other Functions

In addition to the above-mentioned functions, several other roles are attributed to MAAs. Mycosporines can act as metabolites, which regulate the processes of sporulation and germination of fungi, especially among the class *Ascomycetes*, *Basidiomycetes, Deuteromycetes* and *Zygomycetes* [131,132]. They are located in the mucus coat surrounding the fungal conidia, extracellular matrix, and outer layers of the cell wall of microcolonial structures. MAAs are also considered to be regulatory metabolites of reproduction in marine invertebrates [133,134]. Another hypothesis concerns the theoretical role of MAAs as accessory pigments in photosynthesis. It assumes that MAAs are fluorescent compounds that convert UV rays to light utilized for photosynthesis, which increases its efficiency [42,47,80,88,101,111,135]. Nevertheless, this claim has not been verified yet, because MAAs exhibit a low fluorescence, if at all [112,114]. Additionally, several MAAs, such as AS, aplysiapalythine A, B, play a role in ecological connectivity between organisms. MAAs have been described as intraspecific chemical alarm cues for the sea hares *Aplysia californica* and as molecules of keystone significance. The largest concentration of aplysiapalythines A and B has been demonstrated in the defensive secretions and skin of these organisms [98].

## 3. MAAs: A Commercial Approach

### 3.1. Harmful Effects of UV on the Human Body

Human skin is constantly exposed to UVR and exhibits a high sensitivity to its influence [136]. Therefore, ensuring the adequate photoprotection for this largest and one of the most important organs is essential for the proper functioning of the whole body. The exposure to sunlight carries a number of detrimental biological consequences, and these consequences are mainly dependent on the amount of absorbed radiation and the depth of penetration, which is proportional to the incident wavelength. Thus, shorter wavelengths of UV-B are absorbed mostly by keratinocytes of the stratum corneum (SC) to a depth of 160–180 µm, and the longer wavelengths of UV-A penetrate deeper up to approximately 1 mm, thus reaching the dermis layer [137]. Consequently, UV load leads to a wide range of skin damage types. Taking into account the criterion of time between exposure to UV and the occurrence of photoreaction on the skin, skin photolesions are divided into acute and chronic [137,138]. Acute photoreactions on the skin are predominantly generated by overexposure to all fractions of UVR. Alterations typically appear at the site of irradiation within 24 h and are reversible. The most notable alterations include suntan, erythema, edema, blisters, sunburn cells (SBC) formation, phototoxic reactions, photoallergy, photosensitivity and acute photoimmunosuppression [138]. Suntan is one of the visible symptoms of solar irradiation. It is formed by darkening or browning of the melanin pigment in the epidermal layer of the skin. However, it is believed that this process is preceded by previous DNA damage, which stimulates a series of changes leading to the direct oxidation, transfer and synthesis of melanin [139]. There are two types of pigmentation: immediate and late. The first type occurs quickly after UV-A exposure. Melanin precursors are photooxidized to darker melanin, and melanosomes are transferred to keratinocytes. Delayed pigmentation is caused by both UV-A and UV-B within 24 to 72 h, and it involves the stimulation of melanogenesis in melanocytes in the basal layer of the epidermis. The action spectrum of melanogenesis extends from 250 to 400 nm, and its maximum action occurs at 297 nm. Typically, late pigmentation can persist even for several weeks [137,138,140]. Among the other acute skin disorders caused mainly by UV-B rays is erythema. UV-B rays are known as burning rays due to their ability to induce the effect of sunburn 1000 times faster than UV-A. Erythema is an expression of the skin inflammatory process with varying degrees of severity. The skin becomes abnormally reddened, which is a consequence of increased blood flow in connection with the dilatation of superficial vessels. This reaction can be accompanied by a feeling of warmth, pain, burning, general malaise, fever, nausea and headaches. Higher doses of UV can cause edemas (first degree burn) and blisters (second degree burn). Additionally, histological examination of the epidermis reveals the presence of characteristic keratinocytes undergoing apoptosis called SBC. A few days following exposure, erythema regresses spontaneously, leading to darkening and peeling of the skin. Moreover, erythema is regarded as a biomarker of skin photodamages that can stimulate cancer development [137,138,139,140]. One of the most intriguing phenomena caused by UVR is suppression of local or systemic immune response [141,142]. At the cellular level, UVR favours the inhibition of the production of antibodies and immune cells that affect the increase of the body’s sensitivity to various types of infections [143]. In addition, UVR can lead to modifications of morphology and role of the Langerhans cells and can cause enhanced expression of immunosuppressive and anti-inflammatory cytokines (e.g., IL-10) as well as intensive prostaglandin synthesis [144,145,146]. Moreover, UV-B rays absorbed by the SC skin layer stimulate the isomerization of *trans*-urocanic acid to *cis*-urocanic acid [147]. The *cis* isomer has immunosuppressive properties in relation to cellular hypersensitivity and Langerhans cells. UV-A rays can also induce phototoxic reactions, photoallergy and photosensitivity of the skin. In turn, long-term changes in the mammalian skin structure are mainly induced by repetitive and intensive radiation exposure to both UV-B and UV-A. Damage is irreversible and becomes evident after many years. The most important chronic adverse biological effects of UV irradiation include skin-related disorders such as photocarcinogenesis, photoaging and sustained photoimmunosuppression [148,149,150]. UVR is one of the ubiquitous cancer-causing agents [149]. Neoplastic lesions can occur by the direct effect of UV-B and indirect impact of UV-A radiation. UV-B is the most active component of solar light, and its high doses cause disorders at the molecular level. UV-B photons are directly absorbed by biological chromospheres, which are chemical groups of organic molecules, especially by nucleic acids and proteins [151,152,153]. As a consequence of UV-B exposure, these chromospheres undergo conformational changes that disturb their functions, which can lead to a distortion of the course, efficiency and accuracy of many physiological, biochemical and metabolic processes [154,155,156,157]. In the case of keratinocyte DNA, UV-B is absorbed by the purines and pyrimidines representing UV-absorbing chromophores. The main photochemical reactions of these nucleobases proceed by photoisomerization with development of cyclobutane-pyrimidine dimers (CPDs, including thymine dimers) and 6-4 pyrimidine-pyrimidine (6-4-PP) photoproducts [138,140,153,158,159,160,161]. These compounds can aid in the disruption of replication and form deletions and other mutations as well as block RNA transcription. When the cell repair mechanisms fail, the p53 gene is activated, and this gene is responsible for the induction of keratinocyte apoptosis [162]. However, UV-B can cause mutations in this gene that result in the loss of the keratinocyte apoptotic mechanism, which in turn can result in the initiation of epidermal carcinogenesis [139,151,152,163]. Thus, UV-B radiation is considered to be the most important etiologic agent in skin cancer [164]. Prolonged UV exposure of the skin can lead to precancerous changes or chronic neoplastic skin lesions. Skin cancer is derived from the following three main cell types: basal, squamous and melanocytes. The most dangerous and least frequently occurring skin cancer is tumours arising from melanocytes resulting in malignant melanoma [165]. Two other skin cancers, namely squamous and basal cell carcinoma, are more frequent but less aggressive carcinomas [138,140,166]. In opposition to UV-B, UV-A is absorbed by photosensitizers, which readily decompose to form ROS [167,168,169,170,171]. Excess ROS affects the formation of oxidative stress, which contributes to the damage of proteins, lipids and nucleic acids in the skin cells [172,173,174,175,176]. Lipid peroxidation causes the degradation of the cell membrane and loss of cell integrity leading to the loss of skin resilience [169]. Protein oxidation changes the biochemical properties of proteins. In the case of nucleic acids, ROS cause DNA structural modifications by single- and double-strand breaks, and abnormal expression of the genes [176]. Consequently, UV-A indirectly enhances alterations induced by exposure to UV-B. Serious detriments result in cell death [177], whereas minor changes accumulate over years and can lead to the formation of cancer cells. UV-A has been associated with 67% of melanoma cases [178,179]. One of the other symptoms of long-term UV-A exposure is premature skin aging. Thus, this radiation is frequently called aging rays [180]. Persistent UV-A influence can cause progressive deterioration of the dermis structures by the formation of free radicals that in fibroblasts and keratinocytes can elicit activation of matrix metalloproteinases (MMPs). These enzymes are responsible for enhanced degradation of the proteins of extracellular matrix (ECM) that build the skin skeleton, collagen and elastin [181]. As a result, the skin loses elasticity and hardness, thus causing sagging, which accelerates the aging process [180,182]. Exogenous aging is accompanied by the formation of discoloration, spider veins and deepening wrinkles and folds as well as thickening of the SC skin layer, and it is also accompanied by the rough and dry appearance of the skin, widening of the pores and impaired wound healing [182]. The chronic skin lesions include also the sustained UV immunosuppression. UV exposure impairs the effectiveness of the immune system in the fight against tumours by reducing the activity of tumour necrosis factor. This phenomenon significantly supports the growth of melanoma and nonmelanoma cancers [143,148,183].

### 3.2. Internal and External Skin Protection Against UV

A natural skin protection against UV-induced damage is provided by melanin [138]. This dark-coloured pigment accumulates in the form of “umbrella” above the nucleus and is involved in UV absorption and neutralization of the free radicals, protecting keratinocytes DNA against photolesions [138,184]. However, during the excessive exposure to sunlight, the level of its production is low and not sufficient for adequate skin photoprotection. To reduce the risk of UV-induced skin injury, an additional external protection is topically applied, typically in the form of cosmetic products that contain inorganic and organic sunscreens. These filters are able to absorb radiant energy in the range of UV-A and UV-B [185,186]. Inorganic sunscreens are mineral compounds that include zinc oxide and titanium dioxide. On the surface of the epidermis, they form a barrier or “shield” that do not penetrate deep into the epidermis and attenuate UV mainly by absorption superimposed by some scattering [187,188]. They are generally stable and chemically inert, thus do not cause the formation of free radicals or allergic sensitization [8]. In opposition, organic sunscreens are aromatic compounds containing carbonyl groups [89]. They work by absorbing the UVR and converting it into energy [186]. Nevertheless, the ability of some chemical filters, particularly avobenzone, oxybenzone, cinnamates, *para*-aminobenzoic acid (PABA) and its esters, to absorb UVR makes them vulnerable to photolysis with generation of highly reactive products that can penetrate the superficial layers of the epidermis and interact with the cutaneous molecules causing irritation or other photo-sensitizing reactions [20,150,189,190,191]. Organic and inorganic filters in sunscreens show the accumulation potential in the body of water-dwelling organisms, therefore people can be exposed to these compounds also through the food chain [192,193]. Due to the lack of awareness of the above-mentioned threats, people apply chemical sunscreens to the skin frequently, before any exposure to sunlight, believing that it effectively protects their health. Therefore it becomes increasingly important to develop new fully safe for people and environmentally friendly UV filters to address these issues. A promising alternative is the application of multifunctional MAAs, which can be biotechnologically exploited in various ways [12,15,16,20,191,194,195,196].

### 3.3. Potential of MAAs in Skin Protection

#### 3.3.1. MAAs as Sunscreens

In recent studies, MAAs have gained considerable attention as highly active photoprotective candidates for prevention of the harmful effects of UVR on human skin. Oyamada et al. reported that three MAAs, Myc-Gly, SH and PR, extracted from the scallops *Patinopecten yessoensis* ovaries and added to the medium efficiently shielded the WI-38 human lung fibroblasts from UV-induced apoptosis in a dose-dependent manner [109]. Myc-Gly exhibited the strongest photoprotective activity expressed as a half maximal effective concentration (EC_50_) of 24 μM. EC_50_ values for SH and PR were equal to 64 μM and 294 μM, respectively. Moreover, MAAs showed a promotion effect on the TIG-114 normal human skin fibroblasts proliferation. The highest growth-enhancing activity on cells revealed Myc-Gly and SH, which at concentrations of 50 μM augmented fibroblasts proliferation by approximately 35% compared to the untreated control cells. Cell culture experiments conducted by Schmid et al. also demonstrated that the presence of PR and SH from the *P. umbilicalis* in medium stimulated the concentration-dependent growth of the 3T3 mouse fibroblasts exposed to UV-A radiation [197]. Ryu et al. confirmed, in addition, that PR, the most abundant MAA synthetized by the *P. yezoensis*, added at concentrations from 0 to 200 µM to the CCD-986sk human skin fibroblasts culture had no cytotoxic effect on cells viability, and moreover added at concentrations from 0 to 40 µM to fibroblasts previously treated with UVR efficiently protected them against damage in a dose-dependent manner [198]. In contrast, Kim et al. examined the sunscreen effect of 80% methanol extract of the *P. yezoensis*, instead of pure individual MAAs, on the HaCaT human keratinocytes after UV-B irradiation. The treatment with the extract at concentrations of 0.5, 1.0 and 3.0 mg·mL^−1^ following the UV-B exposure significantly enhanced the number of viable cells in the extract concentration-dependent mode, exhibiting the lower degree of increment under the influence of the highest dose of UV-B (70 mJ·cm^−2^) [199]. The authors also showed that the viability of keratinocytes shame-exposed to UVR but treated with the extract increased up to 0.5 mg·mL^−1^, and at higher concentrations has been attenuated. Recent investigations have proved that the protective role of PR against UV-stimulated apoptosis in HaCaT cells involved the suppression of the caspase pathway by lowering the level of a caspase-3 protein [200]. The activity of PR may also relate to the modulation of miRNAs or genes expression patterns associated with UV-affected biological processes such as Wnt (Wingless/integrase-1) and Notch pathways, apoptosis, cell proliferation and translational elongation [201]. Moreover, Ishihara et al. demonstrated that a novel glycosylated MAA, 13-*O*-β-galactosyl-PR, extracted from the cyanobacterium *Nostoc sphaericum* had even higher protective activity on HaCaT culture treated with UV-B and UV-A plus 8-methoxypsoralen induced cell damage than PR [32]. In turn, Torres et al. tested in vitro and in vivo photoprotective effects of a novel MAA, collemin A, isolated from the lichenized ascomycete *Collema cristatum* [29]. In vitro experiment showed that collemin A provided a dose-dependent protection for the HaCaT human keratinocytes against UV-B-promoted cell membrane destruction, while in vivo study where it was diluted to 1:10 in olive oil at a concentration of 6 μg·cm^−2^ completely prevented the formation of erythema, when applied to the human skin 15 min before irradiation. In vivo analysis of the cutaneous UV-protective properties of other MAAs has also been performed by de la Coba et al. for PR and SH isolated from the *P. rosengurttii* [202]. They showed that the galenic formulation containing PR and SH (ratio 88:12) applied topically at a concentration of 4 mg·cm^−2^ to the dorsal skin of the SkhR-1 H female albino hairless mice prevented UV-induced clinical and histophathological damage including erythema, edema, SBC formation, skinfold thickening and other typical structural and morphological alterations observed in non-UV-protected skin biopsies. Additionally, PR and SH counteracted the biochemical changes in UV-exposed skin by maintaining the expression of the heat shock protein Hsp70, a potential biomarker of acute UV damage, and the antioxidant defense system through an effective protection against the strong decrease in the activity of antioxidant enzymes, superoxide dismutase and catalase. In turn, Tosato et al. analysed the direct effect of PR and SH extracted from *P. leucosticta* and incorporated into the Pluronic F-127^®^ polymer gel up to 0.01% weight/volume of MAAs, on the human skin by in vivo confocal Raman spectroscopy [203]. The permeation depth profile of MAAs gel varied within the SC skin layer; the most concentrated was at 2 μm depth with the amount 103.4% higher compared to the outermost layer of SC, and at 4 μm depth decreased by almost 35%. They evaluated the sunscreen efficacy of MAAs formulation by monitoring *trans*-urocanic acid and histidine amount after UV-exposure. The application of MAAs, even at low concentration, prevented UV-induced reduction in the *trans*-urocanic acid and UV-stimulated histidine, maintaining their levels close to normal skin values. In UV-exposed skin treated with the MAAs gel two modes related with *trans* conformation of lipids increased their absorbance compared to normal skin, whereas gauche conformation completely disappeared. Therefore, MAAs formulation effectively protected the human skin against the stress of the natural defense mechanism caused by high doses of UVR. Great efficacy of MAAs as potential sunscreens has also been confirmed by Torres et al., who showed that the crude methanol extract of the cyanobacterium *Aphanizomenon flos-aquae*, rich in PR, exhibited maximum UV-A protection comparable to that specified for the commercial sun care product Boots Soltan Extra Moisturizing Sun Lotion [91]. The extract was characterized by a sun protection factor (SPF) equal to 4, a mean critical wavelength below which 90% of UV absorption occurred equal to 388 nm and a mean ratio of UV-A/UV-B protection factors equal to 0.95. These photoprotection indicators suggest that PR can be one of the compounds providing a wide protection against UVR and serve as UV-A filter. However, Torres et al. recommends that MAAs structures should be slightly modified by replacing the amino acid or amino alcohol moieties by alkyl amino groups to reduce their hydrophilic properties [91].

#### 3.3.2. MAAs as Anti-Cancer Agents

MAAs can also be considered as anti-cancer agents due to their anti-proliferative activities on neoplastic cells. Yuan et al. confirmed that the extracts of red alga, *Palmaria palmata* (dulse), rich in PI, PL, AS, SH and PR exhibited a dose-dependent inhibition of proliferation of the B16-F1 murine skin melanoma cell line [204]. EC_50_ inhibition values for extracts of dulse specimens harvested from locations with low (grade 1) and high (grade 2) UVR intensity after 48 h of incubation were equal to 5.3 and 3.2 mg·mL^−1^, respectively. The greater anti-proliferative efficacy of the grade 2 dulse extract was likely due to the presence of additional MAA, Usu, and reflected its absorption and bioactivity into the murine skin melanoma cells membranes. Moreover, both tested extracts, grades 1 and 2, had comparable the oxygen radical absorbance capacity (ORAC) values which are equal to 36.42 and 38.78 μmol·Trolox·g^−1^ extract, respectively, regardless of the different UV-exposure conditions in locations where dulse specimens were harvested. ORAC activities were likely correlated with the presence of SH and Usu in the extracts. Antioxidant activity of these MAAs can also be involved in the suppression of tumour proliferation. A similar dose-dependent antiproliferative effect of the extracts of wild-harvested (*C. crispus*, *P. palmata*, *Mastocarpus stellatus*) and cultivated (*C. crispus*) red algae containing the same MAAs profile was also revealed against the human HeLa adenocarcinoma cervical and U-937 histiocytic lymphoma cell lines in vitro at concentrations from 0.125 to 4 mg·mL^−1^. Moreover, HeLa cells treated with the extracts of wild *P. palmata* or cultivated *C. crispus* exhibited characteristic apoptotic changes indicating that the antiproliferative activity of these extracts occurs through induction of apoptosis [205]. In turn, Mason et al. revealed the concentration-dependent uptake of SH, the principal UV-absorbing compound in the aqueous extract of red alga *M. stellatus*, by the A431 human epidermal basal carcinoma cells during 48 h exposure in vitro [105]. Interestingly, some MAAs, especially PR, were able to protect DNA molecules in the *P. yezoensis* cells by blocking the UV-dependent production of both CPDs and 6-4-PP in vitro [115]. Also, collemin A at a concentration of 6 μg·cm^−2^ partially protected the irradiated HaCaT human keratinocytes in vitro against pyrimidine dimer formation [29]. Moreover, the prevention of SBC formation in UV-exposed skin by the galenic emulsion with PR and SH applied topically may suggest the contribution of these compounds in DNA protection [202]. Kim et al. also revealed that the MAA abundant *P. yezoensis* extract which has the ability to stimulate apoptosis of UV-damaged HaCaT cells might be an important strategy to prevent development and progression of cancers [199]. Post-treatment of UV-B-exposed (30, 70 mJ·cm^−2^) keratinocytes with the red alga extract at concentrations of 0.5, 1.0 and 3.0 mg·mL^−1^ dose-dependently stimulated an increase of the early and late apoptotic cells fractions, and the increment was proportional to the UV-B doses. Therefore, the extract had a dual impact on the UV-B-exposed HaCaT keratinocytes by enhancing apoptosis of damaged cells while simultaneously inducing overall proliferation and viability of healthy cells. The keratinocytes fate may depend on the extract effect on the changes in redox status and total content of glutathione, the primary cellular antioxidant, under oxidative stress conditions. Cells subjected to the extract treatment at concentrations of 1.0 and 3.0 mg·mL^−1^ showed a dose-dependent increase in the relation of reduced to oxidized glutathione and a decrease in its total content, particularly at the highest concentration. The increase in the apoptotic cell population of damaged keratinocytes can occur via activating the c-Jun N-terminal kinase (JNK) and extracellular signal-regulated kinase (ERK) signalling pathways, in which modulation of a functional system of glutathione can take significant parts. The extract treatment enhanced both the JNK and ERK phosphorylation in UV-B-exposed cells in a dose-dependent manner, but the degree of stimulation of ERK activation was up to 10-fold greater at 3 mg·mL^−1^ compared to JNK. The sham-exposed cells treated with the extract also exhibited a concentration-dependent JNK activating effect, whereas ERK activation was almost negligible. On the other hand, MAAs extract isolated from the edible microalga *Aphanizomenon flos-aquae* at the concentrations up to 5 μM had no significant effect on the human UDP-α-d-glucose 6-dehydrogenase activity, a cytosolic enzyme involved in tumour progression [206].

#### 3.3.3. MAAs as Anti-Photoaging Agents

A few studies also examined the anti-photoaging role of some MAAs. According to in vitro analysis of de la Coba et al., AS, in combination with PI, can effectively reduce the lipid peroxidation that is involved in initiating and/or mediating of the aging process [127]. Also, a mixture of PR and SH, isolated from *P. umbilicalis*, revealed the capacity to suppress UV-A-induced premature aging of human skin in vivo [50,197,207]. Two-week treatment with twice daily application of the formulation containing 0.005% MAAs encapsulated in lecithin liposomes on the inner side of the forearm inhibited the UV-A-stimulated lipid peroxidation by 37%, and four-week treatment significantly improved the skin parameters, firmness, and smoothness, by 10% and 12%, respectively [50,197,207]. Another human in vivo study using similar MAAs preparation shown that it further reduced the depth of wrinkles on the face by almost 20% after 4 weeks of treatment with twice-daily doses [197,207]. Tested MAAs formulation proved to be as efficient as a standard cream with 1% synthetic UV-A filters, Parsol^®^ 1789, and 4% UV-B filters, Neo Heliopan^®^ AV [197]. MAAs ability to prevent extrinsic skin aging was also confirmed in in vitro studies. PR, SH and PI obtained from the dulse and *Porphyra* sp. can serve as anti-skin aging molecules protecting against wrinkles due to their inhibitory properties against MMPs. They showed a dose-dependent inhibition against the bacterium *Clostridium histolyticum* collagenase activity with a half maximal inhibitory concentration values (IC_50_) equal to 104.0 µM, 105.9 µM and 158.9 µM for SH, PR and PI, respectively [208]. Moreover, Ryu et al. revealed that PR at concentrations from 0 to 40 µM efficiently suppressed in a dose-dependent manner the intracellular ROS production and the activity of senescence-associated beta-galactosidase in the UV-A-irradiated CCD-986sk human skin fibroblasts [198]. It also exhibited a concentration-dependent inhibition of UV-A-enhanced MMPs expression; the concentration of 40 µM reduced MMP-1 mRNA expression level even up to 56.2%. Furthermore, PR simultaneously increased the levels of ECM components in UV-A-exposed cells. In the presence of MAA at concentrations of 10, 20 and 40 µM the procollagen secretion levels increased by approximately 23.9%, 16.8%, and 25.1% compared with the non-protected UV-A-irradiated cells, respectively. Similarly, PR enhanced in a dose-dependent manner the expression of type I collagen and elastin as well as blocked their degradation in UV-A-irradiated fibroblasts. It also showed an inhibitory effect on the UV-increased activity of elastase, which leads to elastin decomposition and wrinkles formation; MAA treatment at 10, 20 and 40 µM suppressed the elastase activity by 51.9%, 51.9%, and 82.5% compared with the non-protected UV-A-irradiated cells, respectively. Ryu et al. confirmed that PR has a protective effect on UV-enhanced collagen destruction and can be involved in its synthesis by the negative regulation of MMPs expression and elastinase activity as well as by increasing the secreted procollagen level in UV-damaged human skin fibroblasts [198]. In addition to PR also SH and Myc-Gly isolated from the green algae *Chlamydomonas hedleyi* can modulate the expression of genes related to skin aging, elastin and procollagen, in the UV-irradiated HaCaT human keratinocytes. In the MAAs presence at doses of 0.03 and 0.5 mM, the UV-B-suppressed expression of elastin had significantly enhanced in a dose-dependent way. Similarly, MAAs increased the expression level of the enzyme, procollagen C-endopeptidase enhancer, which bound to the type I procollagen and stimulated the activity of procollagen C-proteinase, but only when SH and PR were applied the increase was concentration-dependent. Additionally, the treatment with Myc-Gly and SH suppressed the UV-induced expression of other gene linked to the aging process, involucrin, which is a marker of keratinocytes differentiation. Moreover, Myc-Gly due to its strong antioxidant properties can block the extrinsic skin aging arising from the UV-induced ROS production. The radical-quenching capacity of Myc-Gly enhanced with a concentration up to 1.5 mM and IC_50_ was equal to 4.23 ± 0.21 mM. Additionally, MAAs can protect the skin against photoaging through the regulation of the expression level of inflammation-related genes, such as COX-2. The treatment of Myc-Gly at concentrations of 0.03, 0.15, or 0.3 mM caused a dose-dependent inhibition of mRNA levels of the COX-2 gene up to about 50% of the control. In contrast, SH at a concentration of 0.03 mM exhibited the anti-inflammatory properties from UV exposure, whereas PR at any concentration studied did not affect the COX-2 expression level. The authors assume that Myc-Gly ability to modulate the UV-stimulated COX-2 expression can be associated with the oxidative process involving the compound [125].

#### 3.3.4. MAAs as Wound Healing Agents

Moreover, MAAs can even act as wound healing agents. Myc-Gly, PR and SH, extracted from the *C. hedlyei* and *P. yezoensis*, at concentrations of 0.1 mg·mL^−1^, 0.05 mg·mL^−1^ and 0.05 mg·mL^−1^, respectively, promoted the wound repair in the HaCaT human keratinocytes on a comparable level as the epidermal growth factor, which typically plays an important role in this process. The molecular mechanism underlying the MAAs-accelerated wound healing in skin cells was associated with the activation of signaling pathways of the focal adhesion kinases (FAK) and mitogen-activated protein kinases (MAPK). The application of MAAs considerably increased the FAK phosphorylation at Y397, which in turn facilitated the activation of MAPKs extracellular signal-regulated kinases (ERK). Cells treated with MAAs were also characterized by the activation of c-Jun N-terminal kinases (JNK), mainly JNK1. Therefore, MAAs likely induce the skin repair by triggering the activation of both kinases, wherein the JNK could be a key player in the process [199].

#### 3.3.5. MAAs as Functional Components of UV-Protective Materials

Recently, MAAs have also gained significant attention due to their potential use as additives to protect non-biological materials such as fabrics, plastics, paints and varnishes against UVR, which affects their properties, durability, quality and lifetime [16,123,209]. Fernandes et al. have developed a new fully natural UV-protective materials consisting of chitosan (CS) as the matrix on which MAAs, Myc-Gly, PR and SH purified from the lichen *Lichina pygmaea*, red algae *P. rosengurttii* and *Gelidium corneum*, respectively, were grafted through amide bond formation based on carbodiimide coupling [210]. The CS-MAA conjugates exhibited a high UV-absorption activity in both UV-A and UV-B regions and the coefficients *ξ* comparable to those determined for the corresponding free MAAs, which are significantly greater than values of UV-protective compounds currently used in sun care products. All three materials showed a high stability under the influence of UVR and temperature of 80 °C for up to 12 h. CS-MAA films pre-incubated independently with culture media for 24 and 48 h have proven to be non-cytotoxic to the L-929 murine fibroblasts. They were biocompatible with cell proliferation, adhesion, and tissue formation. The L-929 cells cultured directly in contact with the different CS-MAA conjugates were able to grow; the highest biological performance assessed based on the proliferation rate, the extent of cell confluence and completeness of adhesion showed CS-Myc-Gly material at 21st day of culture. Additional advantages of constructed films are their biodegradable nature and possibility of their further modifications with other active molecules to produce new multifunctional materials. CS-MAA conjugates have a great potential to use in a variety of biomedical applications to provide an efficient protection against acute and chronic UV-induced skin disorders and to develop the analogues of extracellular matrixes for cell growth and tissue regeneration. Novel biomaterials can be incorporated in wound repair therapy, in the manufacture of artificial skin, artificial cornea and contact lenses, outdoor materials and textiles, food and drug packaging, and coatings.

#### 3.3.6. MAAs Commercial Applications

A formulation developed by Schmid et al. containing the liposomal PR and SH has been commercialized under the name of Helioguard^®^365 and is currently available in the global market [207]. Researchers revealed that apart from a high anti-aging activity, the formulation exhibits protective properties against UV-A-induced loss of cell viability and DNA damage. In vitro studies demonstrated that Helioguard^®^365 added at the concentrations of 0.125% and 0.25% to the HaCaT human keratinocytes exposed to 10 min of UV-A irradiation improved their viability in a dose-dependent manner; the cells viability in the presence of 0.25% Helioguard^®^365 amounted to 97.8%. The addition of 3% and 5% of Helioguard^®^365 to the IMR-90 human fibroblasts irradiated with UV-A range visibly reduced the DNA damage in a concentration-dependent manner. Moreover, Schmid et al. have found that MAAs content in the preparation is stable at 4 °C and room temperature for at least 3 months, while at 37 °C decreases by 20% after this period [207]. Also, 3 months exposure of the formulation to the simultaneous impact of UV-A and various temperatures does not affect the stability of MAAs. Therefore, Helioguard^®^365 exhibits a high preventive effectiveness against UV-A-caused damage to the human skin. Another product offering natural protection against sunburning is Helionori^®^ containing as active ingredients MAAs sunscreens, PI, PR and SH, extracted from the *P. umbilicalis*. The formulation is resistant to solar light exposure by 6 h and to 120 °C by 30 min and stored at a temperature from 15 to 25 °C is stable for at least 18 months. 3 days application of a cream with 5% Helionori^®^ effectively prevented the formation of SBC by 94% compared to an untreated control. Moreover, the formulation exhibited an efficient protective effect on the metabolism of fibroblasts and keratinocytes exposed to UV-A-induced oxidative stress. After 24 h of irradiation in the presence of 2% Helionori^®^ the protection of keratinocytes increased by 57%, and fibroblasts by 135%. The product also provided protection of cellular components against UV-A. 2% Helionori^®^ strongly preserved membrane lipids of keratinocytes by 139% and fibroblasts by 134% as well as offered the maximal protection for DNA [211]. The application potential of MAAs due to their photoprotective and antioxidant activity has also been outlined in a number of patents (Table 1) [17,212]. Interestingly, the discovery of Osborn et al. and Traverso that engineering yeast can efficiently synthesize the natural small-molecule sunscreens opens up the opportunity for their large-scale production for use in the pharmaceutical and cosmetic industries [103,104]. Similarly, their extensive production may take place using an integrated multi-trophic aquaculture (IMTA) system described by Barceló-Villalobos et al. [213]. Moreover, the ability of MAAs to withstand prolonged exposure to UV has prompted researchers to develop based on their UV-absorbing chromophores a new class of synthetic analogues, such as 1-alkyl-3-alcanoyl-1,4,5,6-tetrahydropyridines, which are hydrolytically and oxidatively more stable for commercial use as sunscreens [209,214,215]. Recently, Andreguetti et al. have developed a highly efficient, easy to use and environmentally friendly procedure for the synthesis of nine different MAAs analogues, which can represent a new pathway to obtaining sunscreen agents [216].

## 4. Conclusions

It is becoming clear that MAAs, due to their multiple roles, are commercially attractive compounds. They show a promising future for the application in pharmaceutical and cosmetic industries as natural sunscreens, activators of cells proliferation, anti-cancer agents, anti-photoaging molecules and stimulators of skin renewal. Recently, they have gained much attention due to their ability to use in the manufacture of novel biological and non-biological UV-protective biomaterials. However, a full understanding of pure MAAs effect on the human skin is still far from being complete, and research on the raw extracts are not sufficiently informative due to their changing composition and the synergetic effects between the various constituents.

## Figures and Tables

**Table 1:** Application related patents on MAAs.

Patent Title	Patent Number	References
Extracts of *Aphanizomenon flos aquae* and nutritional, cosmetic and pharmaceutical compositions containing the same	CN 101489527	[217,218,219,220,221,222]
	WO 2008000431	
	CA 2656160	
	MX 2009000137	
	KR 1020090048399	
	US 20100021493	
	US 8337858	
	EP 2032122	
Topical composition comprising transformed bacteria expressing a compound of interest	WO 2014025938	[223,224]
	US 20140044677	
	US 20140044653	
	US 20160000701	
	US 9234204	
Topical formulations for UV protection	WO 2015195546	[225]
Method for producing mycosporine-like amino acid using microbes	WO 2015174427	[226]
Synthesis of UV absorbing compounds	WO 2014082124	[227,228]
	US 20150299124	
UV absorbing compounds, compositions comprising same and uses thereof	WO 2015006803	[229,230]
	US 20160244409	
Imino compounds as protecting agents against ultraviolet radiations	WO 2013181741	[231,232]
	US 20150152046	
Mycosporin-like amino acids, production method thereof, UV protecting agents and antioxidants	JP 2014227339	[233]
Preparation method for laver mycosporine-like amino acids phorphyra-334	CN 102659621	[234]
Beaty product containing desert algae radiation-proof ingredient and natural medical whitening ingredient and preparation method thereof	CN 102764206	[235]
Method for preparing UV screening nontoxic extract from red algae, and nontoxic sunscreen using same	WO 2011096628	[236,237]
	CN 102740869	
Topical composition	WO 2011158041	[238]
Cosmetic sunscreen composition	GB 2472021	[239]
*Aphanizomenon flos aquae* preparation, extracts and purified components thereof for the treatment of neurological, neurodegenerative and mood disorders	WO 2008000430	[240,241,242]
	US 20090311286	
	EP 2046354	
Method for manufacturing non-toxic extract for blocking UV from red algae	KR 100969325	[243]
Mycosporin-like amino acid derivative having glycosyl group and method for producing the same	JP 2009120562	[244]
Sunscreen composition with extract of algae and lichens	ES 2317741	[245]
Compositions comprising *Porphyra* and methods of making and using thereof	WO 2007144779	[246,247]
	US 20070220806	
	EP 2001311	
Uso de aminoácido tipo micosporina (shinorine) en productos para prevención y tratamiento de eritema actínico, fotocarcinogénesis y fotoenvejecimiento	ES 2301426	[248]
Uso de aminoácido tipo micosporina (porfira 334) en productos para prevención de procesos cancerígenos	ES 2301293	[249]
Use of a mycosporin-type amino acid (porphyra 334) as an antioxidant	WO 2007026035	[250]
Use of a mycosporin-type amino acid (M-gly) as an antioxidant	WO 2007026036	[251]
Use of a mixture of mycosporin-type amino acids (asterin 330 + palythine) as an antioxidant	WO 2007026037	[252]
Use of a mycosporin-type amino acid (shinorine) as an antioxidant	WO 2007026038	[253]
Fibroblast growth promoter	JP 2007016004	[254]
Cosmetic including natural substance having sun-screening function	CN 101061995	[255]
Beta-glucuronidase inhibitors for use in deodorants and antiperspirants	US 20040234466	[256,257]
	US 7294330	
Amino-benzophenone UV filter formulations for the prevention of tanning	GB 2412866	[258]
The utilization of natural pigments from lichens, cyanobacteria, fungi and plants for sun protection	WO 2003020236	[259,260,261]
	AU 2002329025	
	EP 1424990	
	US 20050129630	
Cosmetic skin care products and cosmetic agents for protecting skin against premature aging	EP 1473028	[262]
Solar radiation protection composition	WO 2000024369	[263,264,265]
	EP 1123083	
	US 6787147	
Personal care compositions	WO 2002039974	[266,267]
	EP 1341514	
Natural UV filters derived from pigments of lichens	IL 0200725	[268]
Algal extracts containing amino acid analogs of mycosporin are useful as dermatological protecting agents against ultraviolet radiation	FR 2803201	[211]
Topical cosmetic composition, useful for protecting skin and hair against sunlight, contains an extract from the red alga *Polysiphonia lanosa*	FR 2803200	[269]
UV-absorbing compounds and compositions	WO 1990009995	[214]
Sunscreen compositions and compounds for use therein	WO 1988002251	[215]
Mycosporine-like amino acid	JPS 59137450	[270]

## References

[1] Karentz D., McClintock J.B., Baker J. (2001). Chemical defenses of marine organisms against solar radiation exposure: UV-absorbing mycosporine-like amino acids and scytonemin. Marine Chemical Ecology.

[2] Afaq F., Mukhtar H. (2006). Botanical antioxidants in the prevention of photocarcinogenesis and photoaging. Exp. Dermatol..

[3] Rastogi R.P., Sinha R.P. (2009). Biotechnological and industrial significance of cyanobacterial secondary metabolites. Biotechnol. Adv..

[4] Mckenzie R.L., Björn L.O., Bais A., Ilyasd M. (2003). Changes in biologically active ultraviolet radiation reaching the Earth’s surface. Photochem. Photobiol. Sci..

[5] Parisi A.V., Downs N. (2004). Cloud cover and horizontal plane eye damaging solar UV exposures. Int. J. Biometeorol..

[6] Silva A.A. (2015). The diffuse component of erythemal ultraviolet radiation. Photochem. Photobiol. Sci. Off. J. Eur. Photochem. Assoc. Eur. Soc. Photobiol..

[7] Moseley H. (1988). Non-Ionizing Radiation: Microwaves, Ultraviolet and Laser Radiation.

[8] Björn L.O. (2007). Stratospheric ozone, ultraviolet radiation, and cryptogams. Biol. Conserv..

[9] Saika K., Sobue T. (2013). Cancer statistics in the world. Gan To Kagaku Ryoho.

[10] Wang S.Q., Setlow R., Berwick M., Polsky D., Marghoob A.A., Kopf A.W., Bart R.S. (2001). Ultraviolet A and melanoma: A review. J. Am. Acad. Dermatol..

[11] Eisemann N., Waldmann A., Geller A.C., Weinstock M.A., Volkmer B., Greinert R., Breitbart E.W., Katalinic A. (2014). Non-melanoma skin cancer incidence and impact of skin cancer screening on incidence. J. Investig. Dermatol..

[12] Řezanka T., Temina M., Tolstikov A.G., Dembitsky V.M. (2004). Natural microbial UV radiation filters—Mycosporine-like amino acids. Folia Microbiol. Praha.

[13] Sampedro D. (2011). Computational exploration of natural sunscreens. Phys. Chem. Chem. Phys..

[14] Corinaldesi C., Barone G., Marcellini F., Dell’Anno A., Danovaro R. (2017). Marine microbial-derived molecules and their potential use in cosmeceutical and cosmetic products. Mar. Drugs.

[15] Carreto J.I., Carignan M.O. (2011). Mycosporine-like amino acids: Relevant secondary metabolites. Chemical and ecological aspects. Mar. Drugs.

[16] Bandaranayake W.M. (1998). Mycosporines: Are they nature’s sunscreens?. Nat. Prod. Rep..

[17] Richa R., Kumari S. (2011). Biotechnological potential of mycosporine-like amino acids and phycobiliproteins of cyanobacterial origin. Biotechnol. Bioinf. Bioeng..

[18] Eom S.H., Kim S.K., Farris P.K. (2013). Cosmeceutical applications from marine organisms. Cosmeceuticals and Cosmetic Practice.

[19] Sinha R.P., Kim S.K. (2013). Biomedical applications of mycosporine-like amino acids. Marine Microbiology: Bioactive Compounds and Biotechnological Applications.

[20] Bhatia S., Sharma K., Sharma A., Garg A., Kumar S., Purohit A. (2011). Mycosporine and mycosporine-like amino acids: A paramount tool against ultra violet irradiation. Pharmacogn. Rev..

[21] Gröniger A., Sinha R.P., Klisch M., Häder D.P. (2000). Photoprotective compounds in cyanobacteria, phytoplankton and macroalgae—A database. J. Photochem. Photobiol. B Biol..

[22] Shick J.M., Dunlap W.C. (2002). Mycosporine-like amino acids and related gadusols: Biosynthesis, acumulation, and UV-protective functions in aquatic organisms. Annu. Rev. Physiol..

[23] Sinha R.P., Singh S.P., Häder D.P. (2007). Database on mycosporines and mycosporine-like amino acids (MAAs) in fungi, cyanobacteria, macroalgae, phytoplankton and animals. J. Photochem. Photobiol. B Biol..

[24] Dunlap W.C., Shick J.M. (1998). Ultraviolet radiation-absorbing mycosporine-like amino acids in coral reef organisms: A biochemical and environmental perspective. J. Phycol..

[25] Shibata K. (1969). Pigments and a UV-absorbing substance in corals and a blue-green alga living in the Great Barrier Reef. Plant Cell Physiol..

[26] Nakamura H., Kobayashi J., Hirata Y. (1982). Separation of mycosporine-like amino acids in marine organisms using reversed-phase high-performance liquid chromatography. J. Chromatogr..

[27] Singh S.P., Kumari S., Rastogi R.P., Singh K.L., Sinha R.P. (2008). Mycosporine-like amino acids (MAAs): Chemical structure, biosynthesis and significance as UV-absorbing/screening compounds. Indian J. Exp. Biol..

[28] Favre-Bonvin J., Arpin N., Brevard C. (1976). Structure de la mycosporine (P 310). Can. J. Chem..

[29] Torres A., Hochberg M., Pergament I., Smoum R., Niddam V., Dembitsky V.M., Temina M., Dor I., Lev O., Srebnik M. (2004). A new UV-B absorbing mycosporine with photo protective activity from the lichenized ascomycete *Collema cristatum*. Eur. J. Biochem..

[30] Wada N., Sakamoto T., Matsugo S. (2015). Mycosporine-like amino acids and their derivatives as natural antioxidants. Antioxidants.

[31] Klisch M., Richter P., Puchta R., Häder D.P., Bauer W. (2007). The stereostructure of porphyra-334: An experimental and calculational NMR investigation. Evidence for an efficient ‘proton sponge’. Helv. Chim. Acta.

[32] Ishihara K., Watanabe R., Uchida H., Suzuki T., Yamashita M., Takenaka H., Nazifi E., Matsugo S., Yamaba M., Sakamoto T. (2017). Novel glycosylated mycosporine-like amino acid, 13-*O*-(β-galactosyl)-porphyra-334, from the edible cyanobacterium *Nostoc sphaericum*-protective activity on human keratinocytes from UV light. J. Photochem. Photobiol. B Biol..

[33] Carignan M.O., Cardozo K.H.M., Oliveira-Silva D., Colepicolo P., Carreto J.I. (2009). Palythine-threonine, a major novel mycosporine-like amino acid (MAA) isolated from the hermatypic coral *Pocillopora capitata*. J. Photochem. Photobiol. B Biol..

[34] Wu Won J.J., Chalker B.E., Rideout J.A. (1997). Two new UV-absorbing compounds from *Stylophora pistillata*: Sulphate esters of mycosporine-like amino acids. Tetrahedron Lett..

[35] Bohm G.A., Pfleiderer W., Boger P., Scherer S. (1995). Structure of a novel oligosaccharide-mycosporine-amino acid ultraviolet A/B sunscreen pigment from the terrestrial cyanobacterium *Nostoc commune*. J. Biol. Chem..

[36] Carreto J.I., Lutz V.A., De Marco S.G., Carignan M.O., Graneli E., Edler L., Sundström B., Anderson D.M. (1990). Fluence and wavelength dependence of mycosporine-like amino acid synthesis in the dinoflagellate *Alexandrium excavatum*. Toxic Marine Phytoplankton.

[37] Karsten U., Sawall T., Wiencke C. (1998). A survey of the distribution of UV-absorbing substances in tropical macroalgae. Phycol. Res..

[38] Karsten U., Wiencke C. (1999). Factors controlling the formation of UV-absorbing mycosporine-like amino acids in the marine red alga *Palmaria palmata* from Spitsbergen (Norway). J. Plant Physiol..

[39] Rastogi R.P., Sinha R.P., Singh S.P., Häder D.P. (2010). Photoprotective compounds from marine organisms. J. Ind. Microbiol. Biotechnol..

[40] Sinha R.P., Häder D.P. (2008). UV-protectants in cyanobacteria. Plant Sci..

[41] Rastogi R.P., Sinha R.P. (2011). Solar ultraviolet radiation-induced dna damage and protection/repair strategies in cyanobacteria. Int. J. Pharma Bio Sci..

[42] Conde F.R., Churio M.S., Previtali C.M. (2000). The photoprotector mechanism of mycosporine-like amino acids. Excited-state properties and photostability of porphyra-334 in aqueous solution. J. Photochem. Photobiol. B Biol..

[43] Whitehead K., Vernet M. (2000). Influence of mycosporine-like amino acids (MAAs) on UV absorption by particulate and dissolved organic matter in La Jolla Bay. Limnol. Oceanogr..

[44] Carreto J.I., Carignan M.O., Montoya N.G. (2005). A high-resolution reverse-phase liquid chromatography method for the analysis of mycosporine-like amino acids (MAAs) in marine organisms. Mar. Biol..

[45] Callone A.I., Carignan M., Montoya N.G., Carreto J.I. (2006). Biotransformation of mycosporine like amino acids (MAAs) in the toxic dinoflagellate *Alexandrium tamarense*. J. Photochem. Photobiol. B Biol..

[46] Conde F.R., Churio M.S., Previtali C.M. (2007). Experimental study of the excited-state properties and photostability of the mycosporine-like amino acid palythine in aqueous solution. Photochem. Photobiol. Sci. Off. J. Eur. Photochem. Assoc. Eur. Soc. Photobiol..

[47] Cardozo K.H.M., Guaratini T., Barros M.P., Falcão V.R., Tonon A.P., Lopes N.P., Campos S., Torres M.A., Souza A.O., Colepicolo P. (2007). Metabolites from algae with economical impact. Comp. Biochem. Physiol. C Toxicol. Pharmacol..

[48] Sinha R.P., Klisch M., Groniger A., Häder D.P. (2000). Mycosporine-like amino acids in the marine red alga *Gracilaria cornea* - Effects of UV and heat. Environ. Exp. Bot..

[49] Klisch M., Häder D.P. (2008). Mycosporine-like amino acids and marine toxins-The common and the different. Mar. Drugs.

[50] Schmid D., Schürch C., Zülli F. (2004). UV-A sunscreen from red algae for protection against premature skin aging. Cosmetics.

[51] Gröniger A., Häder D.P. (2000). Stability of mycosporine-like amino acids. Recent J. Photochem. Photobiol..

[52] Hirata Y., Uemura D., Ueda K., Takano S. (1979). Several compounds from *Palythoa tuberculosa* (*Coelenterata*). Pure Appl. Chem..

[53] Zhang Z., Gao X., Yuri T., Shingo M., Hiroo O. (2004). Researches on the stability of porphyra-334 solution and its influence factors. J. Ocean Univ. China.

[54] Rastogi R.P., Sonani R.R., Madamwar D., Incharoensakdi A. (2016). Characterization and antioxidant functions of mycosporine-like amino acids in the *Cyanobacterium Nostoc* sp. R76DM. Algal Res..

[55] Yoshiki M., Tsuge K., Tsuruta Y., Yoshimura T., Koganemaru K., Sumi T., Matsui T., Matsumoto K. (2009). Production of new antioxidant compound from mycosporine-like amino acid, porphyra-334 by heat treatment. Food Chem..

[56] Shick J.M., Romaine-Lioud S., Ferrier-Pages C., Gattuso J.P. (1999). Ultraviolet-B radiation stimulates shikimate pathway-dependent accumulation of mycosporine-like amino acids in the coral *Stylophora pistillata* despite decreases in its population of symbiotic dinoflagellates. Limnol. Oceanogr..

[57] Portwich A., García-Pichel F. (2003). Biosynthetic pathway of mycosporines (mycosporine-like amino acids) in the cyanobacterium *Chlorogloeopsis* sp. strain PCC 6912. Phycologia.

[58] Favre-Bonvin J., Arpin N., Brevard C. (1987). Biosynthesis of micosporine: Mycosporine-glutaminol in *Trichothecium roseum*. Phytochemistry.

[59] Singh S.P., Klisch M., Sinha R.P., Häder D.P. (2010). Genome mining of mycosporine-like amino acid (MAA) synthesizing and non-synthesizing cyanobacteria: A bioinformatics study. Genomics.

[60] Balskus E.P., Walsh C.T. (2010). The genetic and molecular basis for sunscreen biosynthesis in cyanobacteria. Science.

[61] Shinzato C., Shoguchi E., Kawashima T., Hamada M., Hisata K., Tanaka M., Fujie M., Fujiwara M., Koyanagi R., Ikuta T. (2011). Using the *Acropora digitifera* genome to understand coral responses to environmental change. Nature.

[62] Tartarotti B., Sommaruga R. (2006). Seasonal and ontogenetic changes of mycosporine-like amino acids in planktonic organisms from an alpine lake. Limnol. Oceanogr..

[63] Rastogi R.P., Incharoensakdi A. (2014). UV radiation-induced biosynthesis, stability and antioxidant activity of mycosporine-like amino acids (MAAs) in a unicellular cyanobacterium *Gloeocapsa* sp. CU2556. J. Photochem. Photobiol. B Biol..

[64] Rastogi R.P., Incharoensakdi A. (2013). UV radiation-induced accumulation of photoprotective compounds in the green alga *Tetraspora* sp. CU2551. Plant Physiol. Biochem..

[65] Taira H., Aoki S., Yamanoha B., Taguchi S. (2004). Daily variation in cellular content of UV-absorbing compounds mycosporine-like amino acids in the marine dinoflagellate *Scrippsiella sweeneyae*. J. Photochem. Photobiol. B Biol..

[66] Klisch M., Häder D.P. (2002). Wavelength dependence of mycosporine-like amino acid synthesis in *Gyrodinium dorsum*. J. Photochem. Photobiol. B..

[67] Laurion I., Roy S. (2009). Growth and photoprotection in three dinoflagellates (including two strains of *Alexandrium tamarense*) and one diatom exposed to four weeks of natural and enhanced ultraviolet-B radiation. J. Phycol..

[68] Neale P.J., Banaszak A.T., Jarriel C.R. (1998). Ultraviolet sunscreens in *Gymnodinium sanguineum* (*Dynophyceae*): Mycosporine-like amino acids protect against inhibition of photosynthesis. J. Phycol..

[69] Riegger L., Robinson D. (1997). Photoinduction of UV-absorbing compounds in Antarctic diatoms and *Phaeocystis antarctica*. Mar. Ecol. Prog. Ser..

[70] Hannach G., Sigleo A.C. (1998). Photoinduction of UV-absorbing compounds in six species of marine phytoplankton. Mar. Ecol. Prog. Ser..

[71] Hernando M., Carreto J.I., Carignan M.O., Ferreyra G.A., Gross C. (2002). Effects of solar radiation on growth and mycosporine-like amino acids content in *Thalassiosira* sp, an Antarctic diatom. Polar Biol..

[72] Kräbs G., Bischof K., Hanelt D., Karsten U., Wiencke C. (2002). Wavelength-dependent induction of UV-absorbing mycosporine-like amino acids in the red alga *Chondrus crispus* under natural solar radiation. J. Exp. Mar. Biol. Ecol..

[73] Franklin L.A., Kräbs G., Kuhlenkamp R. (2001). Blue light and UV-A radiation control the synthesis of mycosporine-like amino acids in *Chondrus crispus* (*Florideophyceae*). J. Phycol..

[74] Singh S.P., Klisch M., Sinha R.P., Häder D.P. (2008). Effects of abiotic stressors on synthesis of the mycosporine-like amino acid shinorine in the cyanobacterium *Anabaena variabilis* PCC 7937. Photochem. Photobiol..

[75] Sinha R.P., Ambasht N.K., Sinha J.P., Klisch M., Häder D.P. (2003). UV-B-induced synthesis of mycosporine-like amino acids in three strains of *Nodularia* (cyanobacteria). J. Photochem. Photobiol. B Biol..

[76] Lesser M.P. (2000). Depth-dependent photoacclimatization to solar ultraviolet radiation in the Caribbean coral *Montastrea faveolata*. Mar. Ecol. Prog. Ser..

[77] Singh S.P., Sinha R.P., Klisch M., Häder D.P. (2008). Mycosporine-like amino acids (MAAs) profile of a rice-field cyanobacterium *Anabaena doliolum* as influenced by PAR and UVR. Planta.

[78] Reef R., Kaniewska P., Hoegh-Guldberg O. (2009). Coral sekeletons defence against ultraviolet radiation. PLoS ONE.

[79] Portwich A., García-Pichel F. (1999). Ultraviolet and osmotic stresses induce and regulate the synthesis of mycosporines in the cyanobacterium *Chlorogloeopsis* PCC 6912. Arch. Microbiol..

[80] Oren A., Gunde-Cimerman N. (2007). Mycosporines and mycosporine-like amino acids: UV protectants or multipurpose secondary metabolites?. FEMS Microbiol. Lett..

[81] Shick J.M., Dunlap W.C., Chalker B.E., Banaszak A.T., Rosenzweig T.K. (1992). Survey of ultraviolet radiation-absorbing mycosporine-like amino acids in organs of coral reef holothuroids. Mar. Ecol. Prog. Ser..

[82] Portwich A., García-Pichel F. (2000). A novel prokaryotic UVB photoreceptor in the cyanobacterium *Chlorogloeopsis* PCC 6912. Photochem. Photobiol..

[83] Korbee Peinado N., Abdala Díaz R.T., Figueroa F.L., Helbling E.W. (2004). Ammonium and UV radiation stimulate the accumulation of mycosporine-like amino acids in *Porphyra columbina* (*Rhodophyta*) from Patagonia, Argentina. J. Phycol..

[84] Litchman E., Neale P.J., Banaszak A.T. (2002). Increased sensitivity to ultraviolet radiation in nitrogen-limited dinoflagellates: Photoprotection and repair. Limnol. Oceanogr..

[85] Llewellyn C.A., Airs R.L. (2010). Distribution and abundance of MAAs in 33 species of microalgae across 13 classes. Mar. Drugs.

[86] Singh S.P., Klisch M., Sinha R.P., Häder D.P. (2010). Sulfur deficiency changes mycosporine-like amino acid (MAA) composition of *Anabaena variabilis* PCC 7937: A possible role of sulfur in MAA bioconversion. Photochem. Photobiol..

[87] Mitchell D.L., Karentz D., Young A., Moan J., Björn L., Nultsch W. (1993). The induction and repair of DNA photodamage in the environment. Environmental UV Photobiology.

[88] Sinha R.P., Klisch M., Gröniger A., Häder D.P. (1998). Ultraviolet-absorbing/screening substances in cyanobacteria, phytoplankton and macroalgae. J. Photochem. Photobiol. B Biol..

[89] Cardozo K.H.M., Carvalho V.M., Pinto E., Colepicolo P. (2006). Fragmentation of mycosporine-like amino acids by hydrogen/deuterium exchange and electrospray ionisation tandem mass spectrometry. Rapid Commun. Mass Spectrom..

[90] Sommaruga R., Whitehead K., Shick J.M., Lobban C.S. (2006). Mycosporine-like amino acids in the zooxanthella-ciliate symbiosis *Maristentor dinoferus*. Protist.

[91] Torres A., Enk C.D., Hochberg M., Srebnik M. (2006). Porphyra-334, a potential natural source for UVA protective sunscreens. Photochem. Photobiol. Sci..

[92] McClintock J.B., Karentz D. (1997). Mycosporine-like amino acids in 38 species of subtidal marine organisms from McMurdo Sound, Antarctica. Antarct. Sci..

[93] Volkmann M., Gorbushina A.A., Kedar L., Oren A. (2006). Structure of euhalothece-362, a novel red-shifted mycosporine-like amino acid, from a halophilic cyanobacterium (*Euhalothece* sp.). FEMS Microbiol. Lett..

[94] Huovinen P., Gómez I., Figueroa F.L., Ulloa N., Morales V., Lovengreen C. (2004). Ultraviolet-absorbing mycosporine-like amino acids in red macroalgae from Chile. Bot. Mar..

[95] Rastogi R.P., Sinha R.P., Moh S.H., Lee T.K., Kottuparambil S., Kim Y.J., Rhee J.S., Choi E.M., Brown M.T., Häder D.P. (2014). Ultraviolet radiation and cyanobacteria. J. Photochem. Photobiol. B Biol..

[96] Helbling E.W., Menchi C.F., Villafañe V.E. (2002). Bioaccumulation and role of UV-absorbing compounds in two marine crustacean species from Patagonia, Argentina. Photochem. Photobiol. Sci..

[97] Newman S.J., Dunlap W.C., Nicol S., Ritz D. (2000). Antarctic krill (*Euphausia superba*) acquire a UV-absorbing mycosporine-like amino acid from dietary algae. J. Exp. Mar. Biol. Ecol..

[98] Kicklighter C.E., Kamio M., Nguyen L., Germann M.W., Derby C.D. (2011). Mycosporine-like amino acids are multifunctional molecules in sea hares and their marine community. Proc. Natl. Acad. Sci. USA.

[99] Davidson A.T. (1998). The impact of UVB radiation on marine plankton. Mutat. Res. Fundam. Mol. Mech. Mutagen..

[100] Holzinger A., Lutz C. (2006). Algae and UV irradiation: Effects on ultrastructure and related metabolic functions. Micron.

[101] Sinha R.P., Häder D.P. (2002). Life under solar UV radiation in aquatic organisms. Adv. Space Res..

[102] Stochaj W.R., Dunlap W.C., Shick J.M. (1994). Two new UV-absorbing mycosporine-like amino acids from the sea anemone *Anthopleura elegantissima* and the effects of zooxanthellae and spectral irradiance on chemical composition and content. Mar. Biol..

[103] Osborn A.R., Almabruk K.H., Holzwarth G., Asamizu S., LaDu J., Kean K.M., Karplus P.A., Tanguay R.L., Bakalinsky A.T., Mahmud T. (2015). De novo synthesis of a sunscreen compound in vertebrates. eLife.

[104] Traverso G. (2015). Why some fish don′t tan. Sci. Transl. Med..

[105] Mason D.S., Schafer F., Shick J.M., Dunlap W.C. (1998). Ultraviolet radiation-absorbing mycosporine-like amino acids (MAAs) are acquired from their diet by medaka fish (*Oryzias latipes*) but not by SKH-1 hairless mice. Comp. Biochem. Physiol. A Mol. Integr. Physiol..

[106] Dunlap W.C., Williams D.M., Chalker B.E., Banaszak A.T. (1989). Biochemical photoadaptation in vision: UV-absorbing pigments in fish eye tissues. Comp. Biochem. Physiol. B.

[107] Przeslawski R., Benkendorff K., Davis A.R. (2005). A quantitative survey of mycosporine-like amino acids (MAAs) in intertidal egg masses from temperate rocky shores. J. Chem. Ecol..

[108] Carefoot T.H., Karentz D., Pennings S.C., Young C.L. (2000). Distribution of mycosporine-like amino acids in the sea hare *Aplysia dactylomela*: Effect of diet on amounts and types sequestered over time in tissues and spawn. Comp. Biochem. Physiol. C Pharmacol. Toxicol. Endocrinol..

[109] Oyamada C., Kaneniwa M., Ebitani K., Murata M., Ishihara K. (2008). Mycosporine-like amino acids extracted from scallop (*Patinopecten yessoensis*) ovaries: UV protection and growth stimulation activities on human cells. Mar. Biotechnol..

[110] Braun C., Reef R., Siebeck U.E. (2016). Ultraviolet absorbing compounds provide a rapid response mechanism for UV protection in some reef fish. J. Photochem. Photobiol. B Biol..

[111] Conde F.R., Churio M.S., Previtali C.M. (2004). The deactivation pathways of the excited-states of the mycosporine-like amino acids shinorine and porphyra-334 in aqueous solution. Photochem. Photobiol. Sci..

[112] Häder D.P., Sinha R.P. (2005). Solar ultraviolet radiation-induced DNA damage in aquatic organisms: Potential environmental impact. Mutat. Res..

[113] Whitehead K., Hedges J.I. (2005). Photodegradation and photosensitization of mycosporine-like amino acids. J. Photochem. Photobiol. B Biol..

[114] Shick J.M., Lesser M.P., Jokiel P.L. (1996). Effects of ultraviolet radiation on corals and other coral reef organisms. Glob. Chang. Biol..

[115] Misonou T., Saitoh J., Oshiba S., Tokitomo Y., Maegawa M., Inoue Y., Hori H., Sakurai T. (2003). UV-absorbing substance in the red alga *Porphyra yezoensis* (*Bangiales*, *Rhodophyta*) block thymine photodimer production. Mar. Biotechnol..

[116] García-Pichel F., Castenholz R.W. (1993). Occurrence of UV-absorbing, mycosporine-like compounds among cyanobacterial isolates and an estimate of their screening capacity. Appl. Environ. Microbiol..

[117] García-Pichel F., Wingard C.E., Castenholz R.W. (1993). Evidence regarding the UV sunscreen role of a mycosporine-like compound in the Cyanobacterium *Gloeocapsa* sp.. Appl. Environ. Microbiol..

[118] Lechowski Z., Białczyk J. (2003). Barwniki ekranujące słoneczne promieniowanie ultrafioletowe u roślin i grzybów [Solar ultraviolet radiation screening pigments in plants and fungi]. Wiadomości Bot..

[119] Ehling-Schulz M., Bilger W., Scherer S. (1997). UV-B-induced synthesis of photoprotective pigments and extracellular polysaccharides in the terrestrial cyanobacterium Nostoc commune.. J. Bacteriol..

[120] Whitehead K., Karentz D., Hedges J. (2001). Mycosporine-like amino acids (MAAs) in phytoplankton, a herbivorous pteropod (*Limacina helicina*), and its pteropod predator (*Clione antarctica*) in McMurdo Bay, Antarctica. Mar. Biol..

[121] Leavitt P.R., Vinebrooke R.D., Donald D.B., Smol J.P., Schindler D.W. (1997). Past ultraviolet radiation environments in lakes derived from fossil pigments. Nature.

[122] Suh H.J., Lee H.W., Jung J. (2003). Mycosporine glycine protects biological systems against photodynamic damage by quenching singlet oxygen with a high efficiency. Photochem. Photobiol..

[123] Dunlap W.C., Yamamoto Y. (1995). Small-molecule antioxidants in marine organisms: Antioxidant activity of mycosporine-glycine. Comp. Biochem. Physiol. B Biochem. Mol. Biol..

[124] Tao C., Sugawara T., Maeda S., Wang X., Hirata T. (2008). Antioxidative activities of a mycosporine-like amino acid, porphyra-334. Fish. Sci..

[125] Suh S.S., Hwang J., Park M., Seo H.H., Kim H.S., Lee J.H., Moh S.H., Lee T.K. (2014). Anti-inflammation activities of mycosporine-like amino acids (MAAs) in response to UV radiation suggest potential anti-skin aging activity. Mar. Drugs.

[126] Bhatia S., Sharma K., Namdeo A.G., Chaugule B.B., Kavale M., Nanda S. (2010). Broad-spectrum sun-protective action of porphyra-334 derived from *Porphyra vietnamensis*. Pharmacogn. Res..

[127] De la Coba F., Aguilera J., Figueroa F.L., de Gálvez M.V., Herrera E. (2009). Antioxidant activity of mycosporine-like amino acids isolated from three red macroalgae and one marine lichen. J. Appl. Phycol..

[128] Kogej T., Gostinčar C., Volkmann M., Gorbushina A.A., Gunde-Cimerman N. (2006). Mycosporines in extremophilic fungi-Novel complementary osmolytes?. Environ. Chem..

[129] Jiang H., Gao K., Helbling E.W. (2008). UV-absorbing compounds in *Porphyra haitanensis* (*Rhodophyta*) with special reference to effects of desiccation. J. Appl. Phycol..

[130] Singh S.P., Häder D.P., Sinha R.P. (2010). Cyanobacteria and ultraviolet radiation (UVR) stress: Mitigation strategies. Ageing Res. Rev..

[131] Sommaruga R. (2001). The role of solar UV radiation in the ecology of alpine lakes. J. Photochem. Photobiol. B Biol..

[132] Arpin N., Bouillant M.L., Turian G., Hohl H.R. (1981). Light and mycosporines. The Fungal Spore: Morphogenetic Controls.

[133] Bandaranayake W.M., Bourne D.J., Sim R.G. (1997). Chemical composition during maturing and spawning of the sponge *Dysidea herbacea* (*Porifera: Demospongiae*). Comp. Biochem. Physiol. B Biochem. Mol. Biol..

[134] Bandaranayake W.M., Des Rocher A. (1999). Role of secondary metabolites and pigments in the epidermal tissues, ripe ovaries, viscera, gut contents and diet of the sea cucumber *Holothuria atra*. Mar. Biol..

[135] Gao K., Wu Y., Li G., Wu H., Villafañe V.E., Helbling E.W. (2007). Solar UV radiation drives CO_2_ fixation in marine phytoplankton: A double-edged sword. Plant Physiol..

[136] Svobodová A., Psotová J., Walterová D. (2003). Natural phenolics in the prevention of UV-induced skin damage. A review. Biomed. Pap..

[137] D’Orazio J., Jarrett S., Amaro-Ortiz A., Scott T. (2013). UV radiation and the skin. Int. J. Mol. Sci..

[138] Brenner M., Hearing V.J. (2008). The protective role of melanin against UV damage in human skin. Photochem. Photobiol..

[139] Schulman J.M., Fisher D.E. (2009). Indoor ultraviolet tanning and skin cancer: Health risks and opportunities. Curr. Opin. Oncol..

[140] Sklar L.R., Almutawa F., Lim H.W., Hamzavi I. (2013). Effects of ultraviolet radiation, visible light, and infrared radiation on erythema and pigmentation: A review. Photochem. Photobiol. Sci..

[141] Kripke M.L. (1984). Immunological unresponsiveness induced by ultraviolet radiation. Immunol. Rev..

[142] Halliday G.M. (2005). Inflammation, gene mutation and photoimmunosuppression in response to UVR-induced oxidative damage contributes to photocarcinogenesis. Mutat. Res..

[143] Gallagher R.P., Lee T.K. (2006). Adverse effects of ultraviolet radiation: A brief review. Prog. Biophys. Mol. Biol..

[144] Simon J.C., Krutmann J., Elmets C.A., Bergstresser P.R., Cruz P.D. (1992). Ultraviolet B-irradiated antigen-presenting cells display altered accessory signaling for T-cell activation: Relevance to immune responses initiated in skin. J. Investig. Dermatol..

[145] Vermeer M., Streilein J.W. (1990). Ultraviolet B light-induced alterations in epidermal Langerhans cells are mediated in part by tumor necrosis factor-alpha. Photodermatol. Photoimmunol. Photomed..

[146] Chung H.T., Burnham D.K., Robertson B., Roberts L.K., Daynes R.A. (1986). Involvement of prostaglandins in the immune alterations caused by the exposure of mice to ultraviolet radiation. J. Immunol..

[147] Capote R., Alonso-Lebrero J.L., García-Pichel F., Brieva A., Pivel J.P., González S. (2006). *Polypodium leucotomos* extract inhibits trans-urocanic acid photoisomerization and photodecomposition. J. Photochem. Photobiol. B Biol..

[148] Cho J.W., Park K., Kweon G.R., Jang B.C., Baek W.K., Suh M.H., Kim C.W., Lee K.S., Suh S.I. (2005). Curcumin inhibits the expression of COX-2 in UVB-irradiated human keratinocytes (HaCaT) by inhibiting activation of AP-1: p38 MAP kinase and JNK as potential upstream targets. Exp. Mol. Med..

[149] Park H.M., Moon E., Kim A.J., Kim M.H., Lee S., Lee J.B., Park Y.K., Jung H.S., Kim Y.B., Kim S.Y. (2010). Extract of *Punica granatum* inhibits skin photoaging induced by UVB irradiation. Int. J. Dermatol..

[150] De Gruijl F.R. (2002). Photocarcinogenesis: UVA vs. UVB radiation. Ski. Pharmacol. Appl. Ski. Physiol..

[151] Besaratinia A., Synold T.W., Chen H.H., Chang C., Xi B., Riggs A.D., Pfeifer G.P. (2005). DNA lesions induced by UV A1 and B radiation in human cells: Comparative analyses in the overall genome and in the p53 tumor suppressor gene. Proc. Natl. Acad. Sci. USA.

[152] Pfeifer G.P., You Y.H., Besaratinia A. (2005). Mutations induced by ultraviolet light. Mutat. Res..

[153] Setlow R.B., Carrier W.L. (1966). Pyrimidine dimers in ultraviolet-irradiated DNA’s. J. Mol. Biol..

[154] Häder D.P., Worrest R.C., Kumar H.D., Smith R.C. (1995). Effects of increased solar ultraviolet radiation on aquatic ecosystems. Ambio.

[155] Sinha R.P., Lebert M., Kumar A., Kumar H.D., Häder D.P. (1995). Spectroscopic and biochemical analyses of UV effects on phycobiliproteins of *Anabaena* sp. and *Nostoc carmium*. Bot. Acta.

[156] Sinha R.P., Singh N., Kumar A., Kumar H.D., Häder M., Häder D.P. (1996). Effects of UV irradiation on certain physiological and biochemical processes in cyanobacteria. J. Photochem. Photobiol. B Biol..

[157] Helbling E.W., Villafrañe V., Ferrario M., Holm-Hansen O. (1992). Impact of natural ultraviolet radiation on rates of photosynthesis and on spedcific marine phytoplankton species. Mar. Ecol. Prog. Ser..

[158] Mitchell D.L. (1988). The relative cytotoxicity of (6–4) photoproducts and cyclobutane dimers in mammalian cells. Photochem. Photobiol..

[159] Mitchell D.L., Nairn R.S. (1989). The biology of the (6–4) photoproduct. Photochem. Photobiol..

[160] Häder D.P., Kumar H.D., Smith R.C., Worrest R.C. (2007). Effects of solar UV radiation on aquatic ecosystems and interactions with climate change. Photochem. Photobiol. Sci..

[161] Katiyar S.K. (2005). Silymarin and skin cancer prevention: Anti-inflammatory, antioxidant and immunomodulatory effects. Int. J. Oncol..

[162] Pustišek N., Šitum M. (2011). UV-radiation, apoptosis and skin. Coll. Antropol..

[163] Trautinger F. (2001). Mechanisms of photodamage of the skin and its functional consequences for skin ageing. Clin. Exp. Dermatol..

[164] Ehrhart J.C., Gosselet F.P., Culerrier R.M., Sarasin A. (2003). UVB-induced mutations in human key gatekeeper genes governing signalling pathways and consequences for skin tumourigenesis. Photochem. Photobiol. Sci..

[165] Moan J., Porojnicu A.C., Dahlback A. (2008). Ultraviolet radiation and malignant melanoma. Adv. Exp. Med. Biol..

[166] Agar N.S., Halliday G.M., Barnetson R.S., Ananthaswamy H.N., Wheeler M., Jones A.M. (2004). The basal layer in human squamous tumors harbors more UVA than UVB fingerprint mutations: A role for UVA in human skin carcinogenesis. Proc. Natl. Acad. Sci. USA.

[167] Wondrak G.T., Jacobson M.K., Jacobson E.L. (2006). Endogenous UVA-photosensitizers: Mediators of skin photodamage and novel targets for skin photoprotection. Photochem. Photobiol. Sci..

[168] Erden Inal M., Kahraman A., Köken T. (2001). Beneficial effects of quercetin on oxidative stress induced by ultraviolet A. Clin. Exp. Dermatol..

[169] Thiele J., Elsner P., Burg G. (2001). Oxidants and antioxidants in cutaneous biology. Current Problem in Dermatology.

[170] Lesser M.P., Farrell J.H. (2004). Exposure to solar radiation increases damage to both host tissues and algal symbionts of corals during thermal stress. Coral Reefs.

[171] Tyrrell R.M., Sies H. (1991). UVA (320–380 nm) as an oxidative stress. Oxidative Stress–Oxidants and Antioxidants.

[172] Dykens J.A., Shick J.M., Benoit C., Buettner G.R., Winston G.W. (1992). Oxygen radical production in the sea anemone *Anthopleura elegantissima* and its endosymbiotic algae. J. Exp. Biol..

[173] Shick J.M., Lesser M.P., Stochaj W.R. (1991). Ultraviolet radiation and photooxidative stress in zooxanthellate *Anthozoa*: The sea anemone *Phyllodiscus semoni* and the octocoral *Clavularia* sp.. Symbiosis.

[174] Lesser M.P., Stochaj W.R. (1990). Photoadaptation and protection against active forms of oxygen in the symbiotic procaryote *Prochloron* sp. and its ascidian host. Appl. Environ. Microbiol..

[175] Lesser M.P. (1996). Acclimation of phytoplankton to UV-B radiation: Oxidative stress and photoinhibition of photosynthesis are not prevented by UV-absorbing compounds in the dinoflagellate *Prorocentrum micans*. Mar. Ecol. Prog. Ser..

[176] Pinnell S.R. (2003). Cutaneous photodamage, oxidative stress, and topical antioxidant protection. J. Am. Acad. Dermatol..

[177] Assefa Z., van Laethem A., Garmyn M., Agostinis P. (2005). Ultraviolet radiation-induced apoptosis in keratinocytes: On the role of cytosolic factors. Biochim. Biophys. Acta Rev. Cancer.

[178] Afaq F., Mukhtar H. (2002). Photochemoprevention by botanical antioxidants. Skin Pharmacol. Appl. Skin Physiol..

[179] Adhami V.M., Syed D.N., Khan N., Afaq F. (2008). Phytochemicals for prevention of solar ultraviolet radiation-induced damages. Photochem. Photobiol..

[180] Krutmann J. (2001). The role of UVA rays in skin aging. Eur. J. Dermatol..

[181] Ichihashi M., Ando H., Yoshida M., Niki Y., Matsui M. (2009). Photoaging of the skin. Anti Aging Med..

[182] Yaar M., Gilchrest B.A. (2007). Photoageing: Mechanism, prevention and therapy. Br. J. Dermatol..

[183] Afaq F., Mukhtar H. (2001). Effects of solar radiation on cutaneous detoxification pathways. J. Photochem. Photobiol. B Biol..

[184] Pathak M.A., Zeise L., Chedekel M.R., Fitzpatrick T.B. (1995). Functions of melanin and protection by melanin. Melanin: Its Role in Human Photoprotection.

[185] González S., Fernández-Lorente M., Gilaberte-Calzada Y. (2008). The latest on skin photoprotection. Clin. Dermatol..

[186] Lowe N.J. (1996). Sunscreens: Development: Evaluation, and Regulatory Aspects.

[187] Osterwalder U., Sohn M., Herzog B. (2014). Global state of sunscreens. Photodermatol. Photoimmunol. Photomed..

[188] Schlossmann D., Shao Y., Shaath N.A. (2005). Inorganic ultraviolet filters. Sunscreens–Regulations and Commercial Development.

[189] Dromgoole S.H., Maibach H.I. (1990). Sunscreening agent intolerance: Contact and photocontact sensitization and contact urticaria. J. Am. Acad. Dermatol..

[190] Nałęcz-Jawecki G., Zawadzki T., Skrzypczak A. (2012). Sunscreens and the environment. Biul. Wydz. Farm. Warsz. Uniw. Med..

[191] Morabito K., Shapley N.C., Steeley K.G., Tripathi A. (2011). Review of sunscreen and the emergence of non-conventional absorbers and their applications in ultraviolet protection. Int. J. Cosmet. Sci..

[192] Giokas D.L., Salvador A., Chisvert A. (2007). UV filters: From sunscreens to human body and the environment. Trends Food Sci. Technol..

[193] Klann A., Levy G., Lutz I., Müller C., Kloas W., Hildebrandt J.P. (2005). Estrogen-like effects of ultraviolet screen 3-(4-methylbenzylidene)-camphor (Eusolex 6300) on cell proliferation and gene induction in mammalian and amphibian cells. Environ. Res..

[194] Raikou V., Protopapa E., Kefala V. (2011). Photo-protection from marine organisms. Rev. Clin. Pharmacol. Pharmacokinet. Int. Ed..

[195] Senevirathne W.S.M., Kim S.K., Dominguez H. (2013). Cosmeceuticals from algae. Functional Ingredients from Algae for Foods and Nutraceuticals.

[196] Bedoux G., Hardouin K., Burlot A.S., Bourgougnon N., Bourgougnon N. (2014). Bioactive components from seaweeds: Cosmetic applications and future development. Advances in Botanical Research.

[197] Schmid D., Schürch C., Zülli F., Nissem H.P., Prieur H. (2003). Mycosporine-like amino acids: Natural UV-screening compounds from red algae to protect the skin against photoaging. SÖFW J..

[198] Ryu J., Park S.J., Kim I.H., Choi Y.H., Nam T.J. (2014). Protective effect of porphyra-334 on UVA-induced photoaging in human skin fibroblasts. Int. J. Mol. Med..

[199] Kim S., You D.H., Han T., Choi E.M. (2015). Modulation of viability and apoptosis of UVB-exposed human keratinocyte HaCaT cells by aqueous methanol extract of laver (*Porphyra yezoensis*). J. Photochem. Photobiol. B Biol..

[200] Suh S.S., Oh S.K., Lee S.G., Kim I.C., Kim S. (2017). Porphyra-334, a mycosporine-like amino acid, attenuates UV-induced apoptosis in HaCaT cells. Acta Pharm..

[201] Suh S.S., Lee S., Youn U., Han S., Kim I.C., Kim S. (2017). Comprehensive expression profiling and functional network analysis of porphyra-334, one mycosporine-like amino acid (MAA), in human keratinocyte exposed with UV-radiation. Mar. Drugs.

[202] De la Coba F., Aguilera J., de Gálvez M.V., Álvarez M., Gallego E., Figueroa F.L., Herrera E. (2009). Prevention of the ultraviolet effects on clinical and histopathological changes, as well as the heat shock protein-70 expression in mouse skin by topical application of algal UV-absorbing compounds. J. Dermatol. Sci..

[203] Tosato M.G., Orallo D.E., Ali S.M., Churio M.S., Martin A.A., Dicelio L. (2015). Confocal Raman spectroscopy: In vivo biochemical changes in the human skin by topical formulations under UV radiation. J. Photochem. Photobiol. B Biol..

[204] Yuan Y.V., Westcott N.D., Hu C., Kitts D.D. (2009). Mycosporine-like amino acid composition of the edible red alga, *Palmaria palmata* (dulse) harvested from the west and east coasts of Grand Manan Island, New Brunswick. Food Chem..

[205] Athukorala Y., Trang S., Kwok C., Yuan Y.V. (2016). Antiproliferative and antioxidant activities and mycosporine-like amino acid profiles of wild-harvested and cultivated edible Canadian marine red macroalgae. Molecules.

[206] Scoglio S., Lo Curcio V., Catalani S., Palma F., Battistelli S., Benedetti S. (2016). Inhibitory effects of *Aphanizomenon flos-aquae* constituents on human UDP-glucose dehydrogenase activity. J. Enzyme Inhib. Med. Chem..

[207] Schmid D., Schürch C., Zülli F. (2006). Mycosporine-like amino acids from red algae protect against premature skin-aging. Euro Cosmet..

[208] Hartmann A., Gostner J., Fuchs J.E., Chaita E., Aligiannis N., Skaltsounis L., Ganzera M. (2015). Inhibition of collagenase by mycosporine-like amino acids from marine sources. Planta Med..

[209] Dunlap W.C., Chalker B.E., Bandaranayake W.M. (1998). Nature’s sunscreen from the Great Barrier Reef, Australia. Int. J. Cosmet. Sci..

[210] Fernandes S.C.M., Alonso-Varona A., Palomares T., Zubillaga V., Labidi J., Bulone V. (2015). Exploiting mycosporines as natural molecular sunscreens for the fabrication of UV-absorbing green materials. ACS Appl. Mater. Interfaces.

[211] Andre G., Pellegrini M., Pellegrini L. (2001). Algal Extracts Containing Amino Acid Analogs of Mycosporin Are Useful as Dermatological Protecting Agents against Ultraviolet Radiation.

[212] Colabella F., Moliné M., Libkind D. (2015). UV sunscreens of microbial origin: Mycosporines and mycosporine-like aminoacids. Recent Pat. Biotechnol..

[213] Barceló-Villalobos M., Figueroa F.L., Korbee N., Álvarez-Gómez F., Abreu M.H. (2017). Production of mycosporine-like amino acids from *Gracilaria vermiculophylla* (*Rhodophyta*) cultured through one year in an Integrated Multi-Trophic Aquaculture (IMTA) system. Mar. Biotechnol..

[214] Chalmers P.J., Fitzmaurice N., Rigg D.J., Thang S.H., Bird G. (1990). UV-Absorbing Compounds and Compositions.

[215] Bird G., Fitzmaurice N., Dunlap W.C., Chalker B.E., Banadaranayake W.M. (1988). Sunscreen Compositions and Compounds for Use Therein.

[216] Andreguetti D., Stein E.M., Pereira C.M.P., Pinto E., Colepicolo P. (2013). Antioxidant properties and UV absorbance pattern of mycosporine-like amino acids analogs synthesized in an environmentally friendly manner. J. Biochem. Mol. Toxicol..

[217] Scoglio S., Canestrari F., Benedetti S., Zolla L. (2016). Extracts of *Aphanizomenon flos aquae* (AFA klamath), Active Compounds, and Their Uses.

[218] Scoglio S., Canestrari F., Benedetti S., Zolla L. (2012). Extracts of *Aphanizomenon flos aquae* and Nutritional, Cosmetic and Pharmaceutical Compositions Containing the Same.

[219] Scoglio S., Canestrari F., Benedetti S., Zolla L. (2010). Extracts of *Aphanizomenon flos aquae* and Nutritional, Cosmetic and Pharmaceutical Compositions Containing the Same.

[220] Scoglio S., Canestrari F., Benedetti S., Zolla L. (2008). Extracts of *Aphanizomenon flos aquae* and Nutritional, Cosmetic and Pharmaceutical Compositions Containing the Same.

[221] Scoglio S., Canestrari F., Benedetti S., Zolla L. (2008). Extracts of *Aphanizomenon flos aquae* and Nutritional, Cosmetic and Pharmaceutical Compositions Containing the Same.

[222] Scoglio S., Canestrari F., Benedetti S., Zolla L. (2014). Extracts of *Aphanizomenon flos aquae* and Nutritional, Cosmetic and Pharmaceutical Compositions Containing the Same.

[223] Qvit-Raz N., Altman T. (2014). Topical Composition Comprising Transformed Bacteria Expressing a Compound of Interest.

[224] Qvit-Raz N., Altman T. (2016). Topical Composition Comprising Transformed Bacteria Expressing a Compound of Interest.

[225] Qvit-Raz N. (2015). Topical Formulations for UV Protection.

[226] Ikeda H., Yamamoto S., Matsumoto J., Sota M. (2015). Method for Producing Mycosporine-Like Amino Acid Using Microbes.

[227] York M., Ryan J. (2015). Synthesis of UV Absorbing Compounds.

[228] Ryan J., York M. (2014). Synthesis of UV Absorbing Compounds.

[229] York M., Ryan J., Savage G.P., Meyer A.G., Jarvis K. (2015). UV Absorbing Compounds, Compositions Comprising Same and Uses Thereof.

[230] York M., Ryan J., Savage G.P., Meyer A.G., Jarvis K. (2016). UV Absorbing Compounds, Compositions Comprising Same and Uses Thereof.

[231] Abou-Khalil E., Raeppel S., Raeppel F. (2013). Imino Compounds as Protecting Agents Against Ultraviolet Radiations.

[232] Abou-Khalil E., Raeppel S., Raeppel F. (2015). Imino Compounds as Protecting Agents Against Ultraviolet Radiations.

[233] Ishihara K., Watanabe R., Suzuki T., Sakamoto T., Matsusato S., Wada N., Takenaka H., Yamaguchi Y. (2014). Mycosporin-Like Amino Acids, Production Method Thereof, UV Protecting Agents and Antioxidants.

[234] Zhang C.H., Xu J.C., Xu Z.H., Gao X. (2012). Preparation Method for Laver Mycosporine-Like Amino Acids Porphyra-334.

[235] Su Z.H., Lin J., Wang F. (2012). Beaty Product Containing Desert Algae Radiation-Proof Ingredient and Natural Medical Whitening Ingredient and Preparation Method Thereof.

[236] Han T.J., Park J.H. (2011). Method for Preparing UV Screening Nontoxic Extract from Red Algae, and Nontoxic Sunscreen Using Same.

[237] Han T.J., Park J.H. (2012). Method for Preparing UV Screening Nontoxic Extract from Red Algae, and Nontoxic Sunscreen Using Same.

[238] O’Connor C., Skill S., Llewellyn C. (2011). Topical Composition.

[239] Wolf F. (2011). Cosmetic Sunscreen Composition.

[240] Scoglio S., Canestrari F., Benedetti S., Benedetti Y., Delgado-Esteban M. (2011). *Aphanizomenon Flos Aquae* Preparation, Extracts and Purified Components Thereof for the Treatment of Neurological, Neurodegenerative and Mood Disorders.

[241] Scoglio S., Canestrari F., Benedetti S., Benedetti Y., Delgado-Esteban M. (2011). *Aphanizomenon Flos Aquae* Preparation, Extracts and Purified Components Thereof for the Treatment of Neurological, Neurodegenerative and Mood Disorders.

[242] Scoglio S., Canestrari F., Benedetti S., Benedetti Y., Delgado-Esteban M. (2008). *Aphanizomenon Flos Aquae* Preparation, Extracts and Purified Components Thereof for the Treatment of Neurological, Neurodegenerative and Mood Disorders.

[243] Han T.J., Park J.H. (2010). Method for Manufacturing Non-Toxic Extract for Blocking UV from Red Algae.

[244] Sakakibara M., Torii M., Miyamoto M. (2009). Mycosporin-Like Amino Acid Derivative Having Glycosyl Group and Method for Producing the Same.

[245] Figueroa F.L., Aguilera J., de la Coba F., Korbee Peinado N. (2010). Sunscreen Composition with Extract of Algae and Lichens.

[246] Ewart H.S., Zhang J., Barrow J.C. (2007). Compositions Comprising *Porphyra* and Methods of Making and Using Thereof.

[247] Ewart H.S., Zhang J., Barrow J.C. (2007). Compositions Comprising *Porphyra* and Methods of Making and Using Thereof.

[248] De la Coba F., Aguilera J., Figueroa F.L. (2008). Uso de Aminoácido Tipo Micosporina (Shinorine) en Productos Para Prevención y Tratamiento de Eritema Actínico, Fotocarcinogénesis y Fotoenvejecimiento.

[249] De la Coba F., Aguilera J., Figueroa F.L. (2009). Uso de Aminoácido Tipo Micosporina (Porfira 334) En Productos Para Prevención de Procesos Cancerígenos.

[250] De la Coba F., Aguilera J., Lopez Figueroa F. (2007). Use of a Mycosporin-Type Amino Acid (Porphyra 334) as An Antioxidant.

[251] De la Coba F., Aguilera J., Figueroa F.L. (2007). Use of a Mycosporin-Type Amino Acid (M-gly) As an Antioxidant.

[252] De la Coba F., Aguilera J., Figueroa F.L. (2007). Use of a Mixture of Mycosporin-Type Amino Acids (Asterin 330 + Palythine) As an Antioxidant.

[253] De la Coba F., Aguilera J., Figueroa F.L. (2007). Use of a Mycosporin-Type Amino Acid (Shinorine) As an Antioxidant.

[254] Ishihara K., Oyamada C., Kaneniwa M. (2007). Fibroblast Growth Promoter.

[255] Kunshang G., Ping L., Juntian X., Zhihui C., Yuming Q. (2007). Cosmetic Including Natural Substance Having Sun-Screening Function.

[256] Banowski B., Hoffmann D., Wadle A., Siegert P., Saettler A., Gerke T. (2004). Beta-Glucuronidase Inhibitors for Use in Deodorants and Antiperspirants.

[257] Banowski B., Hoffmann D., Wadle A., Siegert P., Saettler A., Gerke T. (2007). Beta-Glucuronidase Inhibitors for Use in Deodorants and Antiperspirants.

[258] Baschong W. (2005). Amino-Benzophenone UV Filter Formulations for the Prevention of Tanning.

[259] Enk D.C., Srebnik M., Lev O., Hochberg M., Dor I., Torres A., Dembitsky V.M. (2003). The Utilization of Natural Pigments From Lichens, Cyanobacteria, Fungi and Plants for Sun Protection.

[260] Enk D.C., Srebnik M., Lev O., Hochberg M., Dor I., Torres A., Dembitsky V.M. (2004). The Utilization of Natural Pigments from Lichens, Cyanobacteria, Fungi and Plants for Sun Protection.

[261] Enk D.C., Srebnik M., Lev O., Hochberg M., Dor I., Torres A., Dembitsky V.M. (2005). The Utilization of Natural Pigments from Lichens, Cyanobacteria, Fungi and Plants for Sun Protection.

[262] Schmid D., Schürch C., Zülli F. (2004). Cosmetic Skin Care Products and Cosmetic Agents for Protecting Skin Against Premature Aging.

[263] Krol M., Huner N., Ivanov A., Sarhan F. (2000). Solar Radiation Protection Composition.

[264] Huner N., Krol M., Ivanov A., Sarhan F. (2001). Solar Radiation Protection Composition.

[265] Hunar N., Krol M., Ivanov A., Sarhan F. (2004). Solar Radiation Protection Composition.

[266] Llewellyn C., Galley E. (2002). Personal Care Compositions.

[267] Llewellyn C., Galley E. (2003). Personal Care Compositions.

[268] Enk D.C., Hochberg M., Torres A., Lev O., Dor I., Dembitsky V.M., Srebnik M. (2002). Natural UV Filters Derived from Pigments of Lichens.

[269] Sirop J.C., Pradines R.D. (2001). Topical Cosmetic Composition, Useful for Protecting Skin and Hair Against Sunlight, Contains an Extract From the Red Alga Polysiphonia lanosa.

[270] Nakamura E., Kobayashi J., Abe R. (1984). Mycosporine-Like Amino Acid.

